# Binding of high mobility group A proteins to the mammalian genome occurs as a function of AT-content

**DOI:** 10.1371/journal.pgen.1007102

**Published:** 2017-12-21

**Authors:** Daniele F Colombo, Lukas Burger, Tuncay Baubec, Dirk Schübeler

**Affiliations:** 1 Friedrich Miescher Institute for Biomedical Research, Basel, Switzerland; 2 Faculty of Science, University of Basel, Basel, Switzerland; 3 Swiss Institute of Bioinformatics, Basel, Switzerland; Edinburgh Cancer Centre, UNITED KINGDOM

## Abstract

Genomic location can inform on potential function and recruitment signals for chromatin-associated proteins. High mobility group (Hmg) proteins are of similar size as histones with Hmga1 and Hmga2 being particularly abundant in replicating normal tissues and in cancerous cells. While several roles for Hmga proteins have been proposed we lack a comprehensive description of their genomic location as a function of chromatin, DNA sequence and functional domains. Here we report such a characterization in mouse embryonic stem cells in which we introduce biotin-tagged constructs of wild-type and DNA-binding domain mutants. Comparative analysis of the genome-wide distribution of Hmga proteins reveals pervasive binding, a feature that critically depends on a functional DNA-binding domain and which is shared by both Hmga proteins. Assessment of the underlying queues instructive for this binding modality identifies AT richness, defined as high frequency of A or T bases, as the major criterion for local binding. Additionally, we show that other chromatin states such as those linked to cis-regulatory regions have little impact on Hmga binding both in stem and differentiated cells. As a consequence, Hmga proteins are preferentially found at AT-rich regions such as constitutively heterochromatic regions but are absent from enhancers and promoters arguing for a limited role in regulating individual genes. In line with this model, we show that genetic deletion of Hmga proteins in stem cells causes limited transcriptional effects and that binding is conserved in neuronal progenitors. Overall our comparative study describing the *in vivo* binding modality of Hmga1 and Hmga2 identifies the proteins’ preference for AT-rich DNA genome-wide and argues against a suggested function of Hmga at regulatory regions. Instead we discover pervasive binding with enrichment at regions of higher AT content irrespective of local variation in chromatin modifications.

## Author summary

We investigated the chromosomal location of a group of highly abundant nuclear proteins. Our genome-wide results for Hmga1 and Hmga2 reveal a unique binding modality indicating preference for DNA rich in A or T bases *in vivo*. Importantly this preferential binding to AT-rich sequences occurs throughout the genome irrespectively of other local chromatin features. Genomic location and loss of function experiments challenge the view that Hmga proteins act as local modulators of transcriptional regulation but rather argue for a role as structural components of chromatin.

## Introduction

With the advent of genomics techniques, the understanding of the many roles of histone proteins and their modifications has increased rapidly [1,2]. However, comparably little attention has been given to the second most abundant class of nuclear proteins after histones [3], the high mobility group proteins [4].

Initially described as small proteins (< 30 KDa) associated with chromatin [5], they were named after their fast mobility in polyacrylamide gels. In mouse and humans, high mobility group proteins are highly conserved and have been divided into 3 families (A, B, N) based on their different structural features [6]. The two members of the A group, Hmga1 and Hmga2, are ~100 amino acids (AA) long intrinsically disordered proteins, which possess 3 DNA-binding domains (DBD) and a short acidic tail [7]. The DBDs are constituted by short stretches of positively charged amino-acids that contact the minor groove of the DNA [8]. Compared to Hmga1, Hmga2 harbors a smaller linker between the first and the second DBD and a longer AA sequence between the third DBD and the acidic tail. Nevertheless, within the 3 DBDs there is high conservation between both proteins with 74% identity and 15% similarity (S1A Fig).

Hmga proteins are robustly expressed during embryonic development and in rapidly replicating cells (such as hematopoietic lineages) [9,10] but have been found misregulated and/or truncated in a number of cancers [11–14]. Whereas expression of Hmga1 is upregulated in hematopoietic malignancies [15], Hmga2 overexpression has been associated with malignant epithelial tumors [16,17]. The Hmga2 gene has also been linked to rearrangements, mostly in benign tumors of mesenchymal origin [14]. However, while increasing evidence indicates that deregulation and rearrangements of Hmga proteins are present both in malignant and benign neoplasia, Hmga overexpression also seems to sensitize cancerous cells to various genotoxic agents [6].

From a functional perspective, Hmga proteins have been mainly implicated in regulating chromatin architecture through direct interaction with histone proteins or with the transcriptional machinery [10]. Additional mechanisms for a role in transcriptional control range from stabilization of enhancer-associated protein complexes through displacement of positioned nucleosomes (for the activation of IFN-beta and IL-2Ralpha genes) [18,19], to competition with histone H1 [20], to direct interaction with the mediator complex [21] and histone chaperones [22]. In light of the disparate physiological functions described, many mechanisms have been proposed that link tumorigenesis and malignant transformation with Hmga misregulation [23].

Such functional models make predictions on the chromosomal location of Hmga proteins and suggest preferential binding to regulatory regions. Attempts to determine genomic localization have however remained limited. The Hmga1 protein was discovered due to its ability to bind *in vitro* to a primate major satellite sequence [24] and its DBD was called AT-hook since DNA sequences protected from footprinting were rich in A or T nucleotides. Many studies replicated Hmga1-2 binding to AT-rich DNA (reviewed in [20]), however such studies mainly focused on single loci experiments and were mostly conducted *in vitro*.

Regarding the *in vivo* genome-wide binding determination of Hmga proteins, data is only available for Hmga2. In the first *in vivo* determination of Hmga2 binding preference, the identified motif (consensus of 49 sequences) was a simple repetition of W nucleotides (either A or T) [25]. This is in contrast with a low-throughput SELEX assessment of Hmga2 affinity, which resulted in a high-information-content DNA logo [26]. In another study, a ChIP-chip experiment was performed in the MKN28 gastrinoma cell line overexpressing Hmga2 [27], yet binding preferences were not discussed in this work. Similarly, a recently published Hmga2 ChIP-seq study in mouse embryonic fibroblasts [28] did not comment on sequence specificity while reporting promoter-centered enrichments. This pattern however, warrants caution as promoters are sites of open chromatin, which frequently causes an intrinsic bias in ChIP-seq experiments [29]. We reasoned that a more thorough understanding of the mechanism adopted *in vivo* for DNA and chromatin recognition by Hmga proteins will help shed light on the many, partially opposing functions that have been described.

Here we use an antibody-free ChIP-seq approach to investigate the location of Hmga1-2 proteins genome-wide. We adopted a flexible cellular system in mouse embryonic stem cells that allows stereotyped expression of different protein constructs. This reveals widespread DNA-binding throughout the genome, with a preference for DNA with high AT content. Interestingly this binding appears neither affected by chromatin state, nor linked to regulatory regions and is conserved upon neuronal differentiation, underscoring the robustness of the primary sequence readout. Accordingly regions that show a compositional bias towards AT, like heterochromatin found at major satellites [30], show on average high occupancy of both Hmga proteins.

## Results

### Unbiased genome-wide location analysis of Hmga proteins

**Fig 1:**
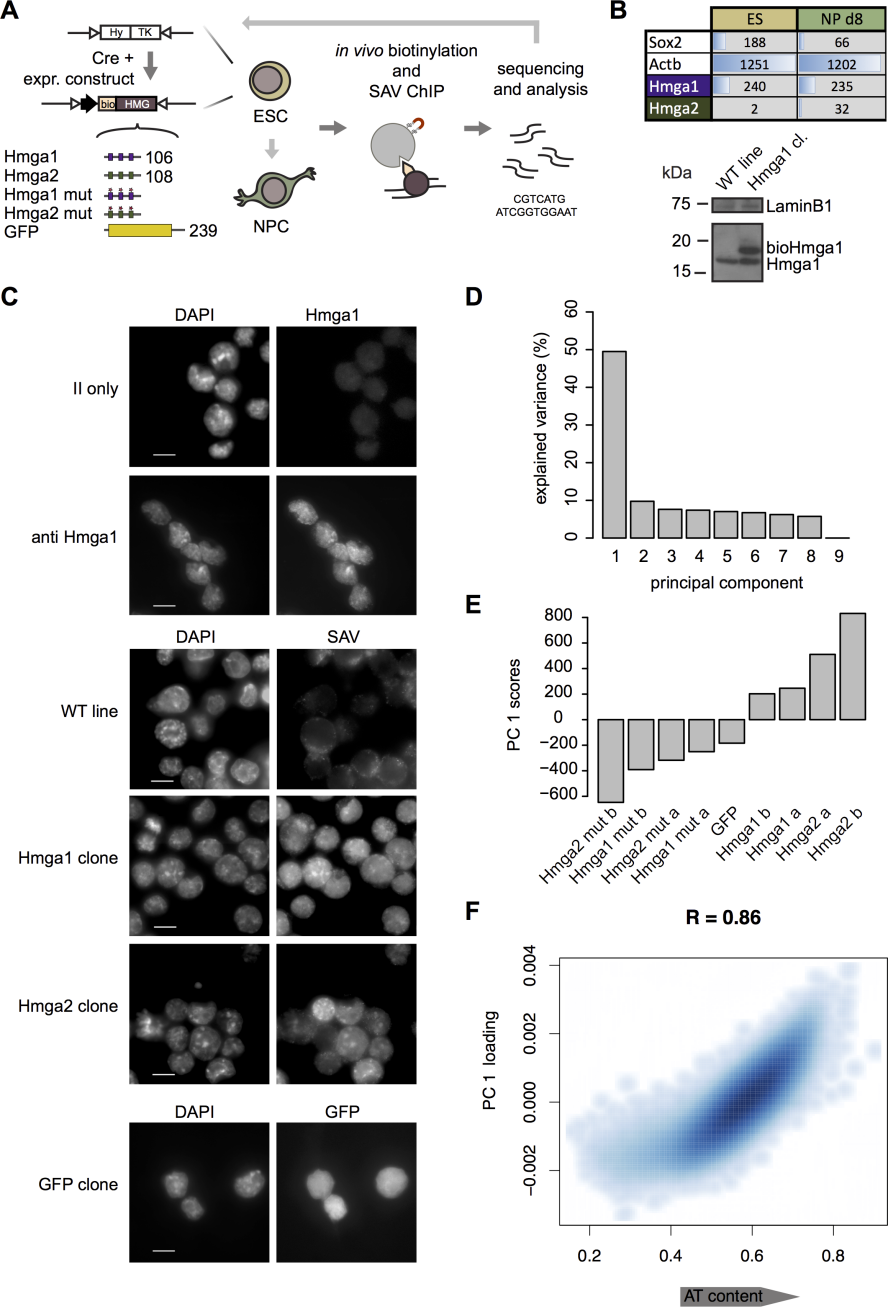
Genomic location of Hmga1-2 versus DBD-mutant controls. (A) Biotin-tagged versions of Hmga proteins driven by a strong, ubiquitously active promoter were inserted into a defined genomic locus. DBDs of Hmga1-2 are depicted as boxes. Mutations in the DBD of Hmga1-2 where targeted to the core RGR motif of the three AT-hooks. A monomeric GFP control was tagged and inserted in a similar way. The N-terminal biotin tag is recognized by the BirA biotin ligase, which the cell line used stably expresses. Subsequent streptavidin (SAV) mediated Chromatin-IP followed by sequencing was used to generate antibody-independent genomic maps. Functional mutants were similarly expressed after insertion into the same genomic location. (B) Top, table shows read counts per kilobase and million mapped reads (RPKM) for Hmga1, Hmga2 and two control genes. To account for an Hmga1 pseudogene, mapping was performed allowing 20 multiple alignments and reported values are likely an underestimation of actual expression levels. Hmga2 is not expressed in ES cells. Bottom, Western blotting (WB) with anti Hmga1 Ab of whole cell lysate from parental cell line and cells expressing Hmga1. A higher molecular weight band representing bioHmga1 is visible and shows an expression level comparable to the endogenous protein NP stands for neuronal progenitors. (C) Subcellular localization of endogenous Hmga1, top set, bioHmga proteins, middle set, and bioGFP, bottom set, assessed by immunofluorescence. Nuclei and DNA were stained with DAPI. DAPI-dense foci are positive for both WT and tagged Hmga proteins detected with a specific antibody and with SAV-coupled fluorophores, respectively. The subcellular localization of tagged GFP by GFP-channel acquisition is also depicted. GFP stains evenly in ESC and is not excluded from nuclei. Scale-bars in the DAPI channel corresponds to 10 μm. (D) Log2 enrichments over input in 1 kb tiling windows of DBD mutant and WT Hmga1-2 as well as GFP were subjected to Principal Component Analysis (PCA). Barplot shows fraction of the total variance explained by each principal component. The first principal component (PC1) alone explains almost 50% of the variance. (E) PC1 scores of each sample. PC1 separates the samples into those corresponding to proteins with a WT DNA-binding domain and those with either a mutated or no DBD. (F) Scatterplot and Pearson correlation of the PC1 loading with AT content.

We aimed to investigate the *in vivo* DNA and chromatin-binding preferences of Hmga1 and Hmga2 proteins. Given the discordant results obtained using antibodies [26,28], we decided to determine their genomic location with our previously established RAMBiO approach, a biotin-tagging protocol that combines stringent ChIP washes with controlled transgene expression and the possibility to measure genomic binding in stem and differentiated cells [31,32]. Importantly, the approach also allows an assessment of the binding preferences of functional mutants under identical experimental conditions, either a priori or after analysis of the wild-type (WT) data (Fig 1A).

We designed recombination constructs for the main isoforms of Hmga1 and Hmga2, respective DBD-mutants and a GFP control, each flanked by inverted lox sites to enable site-specific targeting by the Cre recombinase. Utilizing this comprehensive sample set enabled unambiguous assessment of whether genomic location was a reflection of genuine DNA binding. As DBD-mutants we generated Hmga variants that are mutated at the conserved arginines of the central Arg-Gly-Arg motif of the DBDs (S1A Fig), previously shown to be important for DNA binding *in vivo* [33]. As a control for unspecific interaction with DNA we utilized monomeric GFP, which is known to diffuse freely in the cellular volume [34].

After transfection, individual cell clones of mouse embryonic stem cells (ESC) were isolated and characterized for targeted integration at a previously utilized chromosomal location that confers stable and homogeneous expression [32]. RNA-seq expression profiling showed that Hmga1 is expressed to levels comparable to a master transcription factor (i.e. Sox2 in ESC) and that Hmga2 is not expressed at the stem cell stage (Fig 1B, top). However, Hmga2 expression increases during differentiation towards a neuronal lineage (Fig 1B, top) and the Hmga2 protein can be detected at the neuronal progenitor stage (S1B Fig). Protein quantification by Western Blotting revealed that the tagged Hmga1 is expressed at levels comparable to endogenous Hmga1 (Fig 1B, bottom). Tagged Hmga2 is expressed similarly to Hmga1 after insertion into the same genomic locus and under the same promoter (S1C Fig). Importantly, in our cellular system, introduction of either bioHmga1 or bioHmga2 (unless otherwise specified, abbreviated as Hmga1 and Hmga2 from hereon) did not result in any apparent change in clonogenic potential (S1D Fig). Hmga1 and Hmga2 DBD-mutants were similarly quantified and levels were comparable to the respective tagged WT proteins (S1E and S1F Fig).

Next we determined the subcellular localization of Hmga1-2 proteins by immunofluorescence. In line with previous observations [14,35], we detected enrichment at DAPI-dense foci for both the biotin-tagged proteins and endogenous Hmga1 (Fig 1C, middle and top set respectively). Colocalization of the signal was also present between the tagged and the endogenous Hmga1, pointing to a complete functional equivalence (S1G Fig). Tagged monomeric GFP was distributing throughout the cell volume (Fig 1C, bottom set), as previously described for monomeric GFP [34].

In light of the correct subcellular localization of the biotinylated proteins we proceeded with bioChIP. After pull-down, a considerable amount of DNA was retrieved for both Hmga proteins (up to 1/500 of the total amount of DNA subjected to chromatin IP), potentially hinting at a high intrinsic affinity for chromatin. Interestingly, we noted that for DBD-mutants much less DNA was recovered (down to 1/10’000 of the total amount of DNA subjected to chromatin IP), indicating that mutations in the DBD indeed compromised the ability to bind chromatin.

In order to stringently account for systematic biases, we generated paired input controls for each IP condition. Additionally, we used similar numbers of PCR cycles for both IP and input chromatin during library preparation in order to minimize biases arising from different rounds of exponential amplification [36]. Subsequent next-generation sequencing and input normalization revealed good reproducibility but, upon visual inspection, a lack of focal sites of binding as previously observed for binders of low complexity motifs such as MBD proteins [32] or DNA methyltransferases [31]. Systematic analysis of Hmga binding behaviour thus requires direct comparison of binding between WT proteins and DBD-mutant to rigorously identify sequence or chromatin features that direct binding.

As a first step, we performed principal component analysis (PCA) on log2 enrichments of IP over input for two replicates of WT proteins, DBD-mutants, and a GFP sample, calculated over 1kb tiling windows along the genome. Strikingly, the first principal component (PC1) accounts for almost 50% of the total variance in the data (Fig 1D) while none of the other principal components explain more than 10%, suggesting that the main signal in the data is contained in PC1. In agreement with a direct DNA-binding modality of Hmga proteins, the PC1 scores revealed a clear separation between WT Hmga proteins and GFP or DBD-mutant proteins which was reproducible among replicates (Fig 1E). To directly link PC1 to physical variables, we contrasted the PC1 loading to marks of chromatin states and genomic features (S1H Fig). This revealed that, while the PC1 loading was only moderately related to chromatin features (|R| ≤ 0.43), it was highly correlated (R = 0.86) to AT content, a metric of DNA compositional bias (Fig 1F). AT content is simply 1—GC-content and can be directly calculated as the percentage of A and T nucleotides over regions of DNA, in this case and throughout the manuscript (unless otherwise specified) 1-kb genomic windows. This result suggests that AT content alone can instruct functional Hmga1-2 binding.

### Genomic binding to AT-rich DNA as a function of the DNA-binding domain

**Fig 2:**
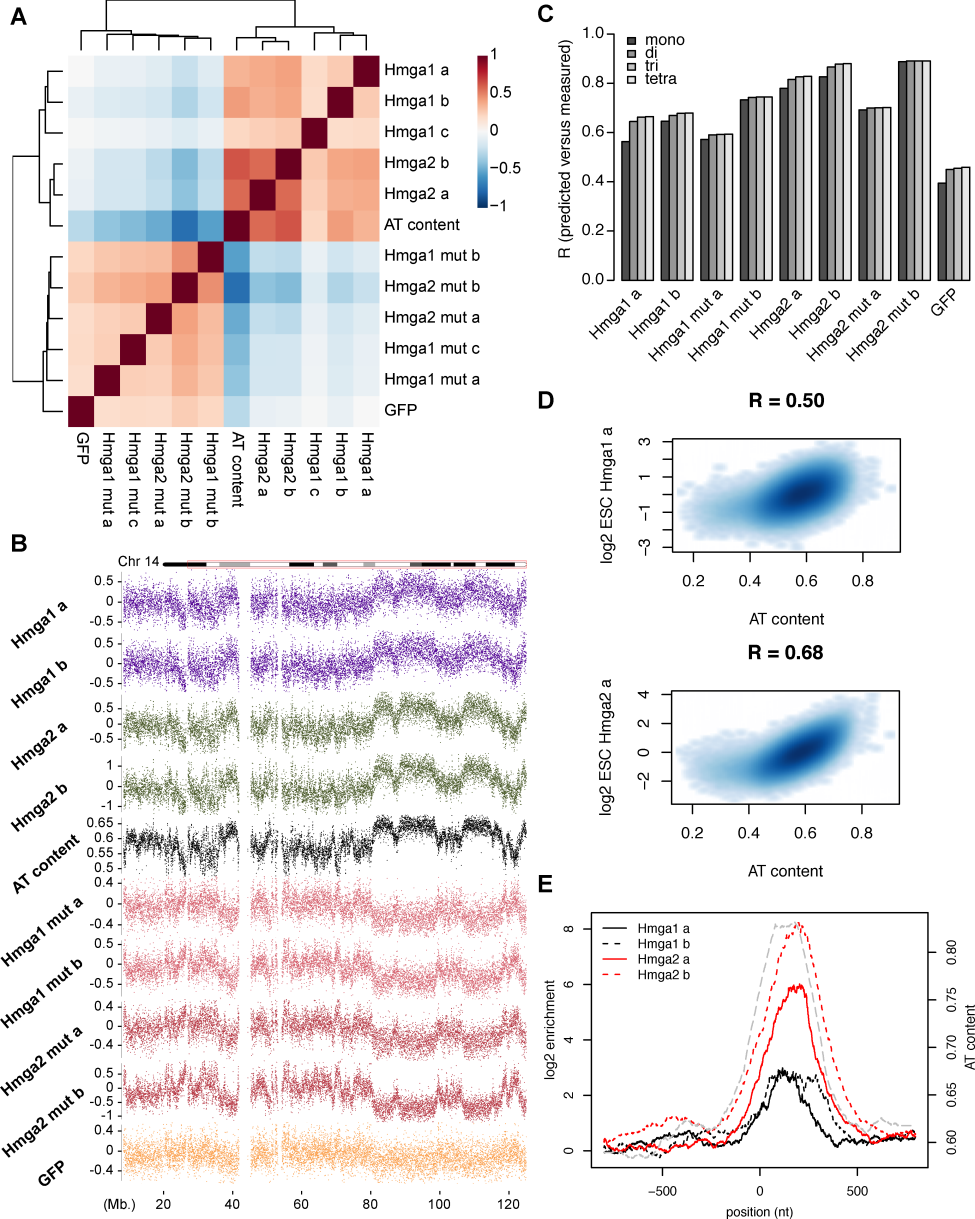
Hmga proteins bind to DNA in ESC as a function of DNA AT content. (A) Genome-wide correlation heatmap for all samples (including replicate c of Hmga1) and AT content on 1kb tiling windows, illustrating both good reproducibility between replicates and the correlation of Hmga1-2 with AT content. Colors indicate the Pearson correlation coefficient. (B) Log2 enrichments over input for the depicted samples on chromosome 14. Each dot represents the enrichment of IP over input in a window of size 10kb. Gaps indicate regions with low mappability (below 80%). Top and bottom 1% of data range are not shown to enhance readability. (C) Barplot illustrating the predictive power of mono-, di-, tri- and tetranucleotide models for each individual sample. The predictive power is not substantially improved by taking into account higher-order sequence features. (D) Relationship between bioHmga samples and AT content in ES cells for two representative replicates. Scatterplots depict AT content vs. log2 Hmga1-2 input-normalized enrichment values (over 1kb tiling windows) minus the same enrichments for the respective DBD-mutant. Pearson correlation coefficients are indicated on top. (E) Hmga binding at simple repeats with very high AT content. Average profiles show log2 enrichment over the respective DBD mutants at mappable (TA)n simple repeats of a minimal length of 300nts, centered at repeat start coordinates. AT content is shown in grey (dashed line).

To further explore the link to AT content, we directly performed hierarchical clustering of input normalized data from all samples and AT content. This identifies one cluster which groups WT samples and their replicates together with AT content, and a second cluster consisting of the DBD-mutant and GFP control samples (Fig 2A). The robustness of this observation was explicitly confirmed by a repetition of the bioChIP-seq experiments using different buffers for cell lysis and SAV precipitation (replicates c in Fig 2A and S2A Fig).

Since at megabase scale, the genome shows clear structures in terms of AT content due to the presence of isochores [37,38], Hmga1-2 differential binding is best visually appreciated by plotting enrichments over an entire chromosome (Fig 2B). This reveals broad regions of high and low enrichment that are shared between Hmga1 and Hmga2 (Fig 2B, in purple and green), consistent between replicates (Fig 2B and S2B Fig) and that follow AT content. Interestingly, the GFP signal, albeit noisy, resembles the binding profile of the DNA-binding mutants of Hmga1-2 (Fig 2A and 2B, yellow track). Importantly, interaction of the DBD-mutants with DNA is increased at regions where Hmga enrichment is low (lower tracks in Fig 2B) providing direct support for the notion that Hmga1-2 binding is specific. Additionally, since only the DNA-binding domain was altered in the mutants, this data further argues that no other protein domain contributes to genomic binding. Together, these extensive controls show that binding of Hmga proteins *in vivo* is variable between different genomic regions but dependent on a functional DNA-binding domain.

To further dissect the nature of the AT dependence, we tested whether specific AT-rich motifs were preferentially bound by Hmga1 and Hmga2 proteins. As the absence of focal binding prevents a motif finding-based analysis, we instead, using ridge regression, modelled signal dependence in 1kb tiling windows as a function of nucleotide frequencies of increasing complexity (mono-, di-,tri- and tetranucleotides, see Materials and methods and S3A Fig for details). The inferred coefficients for the mononucleotide model again confirm the importance of A or T nucleotides (S3B Fig). Interestingly, the improvement in predictive power obtained by accounting for higher-order sequence combinations compared to the mono-nucleotide model is only modest (Fig 2C and S3C–S3E Fig). Similarly, longer stretches of As and/or Ts appear not to create binding sites that are more strongly bound than predicted by the mononucleotide preference (S4A and S4B Fig). Next we asked if local DNA shape, which varies based on combinations of neighbouring bases [39], can improve the mononucleotide model. At the resolution allowed by our study, including DNA shape leads to only minor improvements in predictive power suggesting limited influence on binding (S4C Fig).

We thus propose that binding of Hmga proteins to genomic DNA occurs as a function of AT content alone and is not noticeably increased when specific DNA-sequence motifs are present. This binding behaviour directly accounts for the lack of focal enrichments of Hmga proteins since As and Ts are inherently abundant in DNA even though they vary in frequency. This is readily illustrated at CpG islands where AT content drops sharply and on average by ~20%. This coincides with a local decrease in Hmga binding (S2C Fig).

Since Hmga1 and 2 are independently regulated in our model system and expressed with different tissue specificity [40,41], we next asked for potential variation in the strength of AT-dependence between proteins, which could indicate non-redundancy of their function. For this and for all subsequent analyses, we further normalized the log2 WT enrichment values by subtracting the log2 enrichment values of the respective DBD-mutant (see Materials and methods). We reasoned that this metric would be more accurate for a thorough description of Hmga1 and Hmga2 DNA-binding activity since it accounts for sequencing bias and unspecific binding. This comparison reiterates the positive correlation between binding and AT content for both proteins and a stronger AT-dependence for Hmga2 (Fig 2D). Despite this small difference, AT-dependence appears similar for both proteins and thus functional differences, if any, should probably be ascribed to different interaction partners and not to differences in the DNA-binding readout. Motivated by this AT dependence we determined the binding to extremely AT-rich repeats (roughly 80% AT) that are of sufficient length (at least 300 bps, similar to ChIP assay resolution) and still mappable to specific sites. A subgroup of (TA)n simple repeats fulfills these criteria and these indeed show strong binding (Fig 2E), and are in several cases even visible as peaks at the single locus level (S2D–S2F Fig). This again argues that AT content is an important contributor to Hmga binding.

### Chromatin-feature independence and AT-content dependence in a different cell type

**Fig 3:**
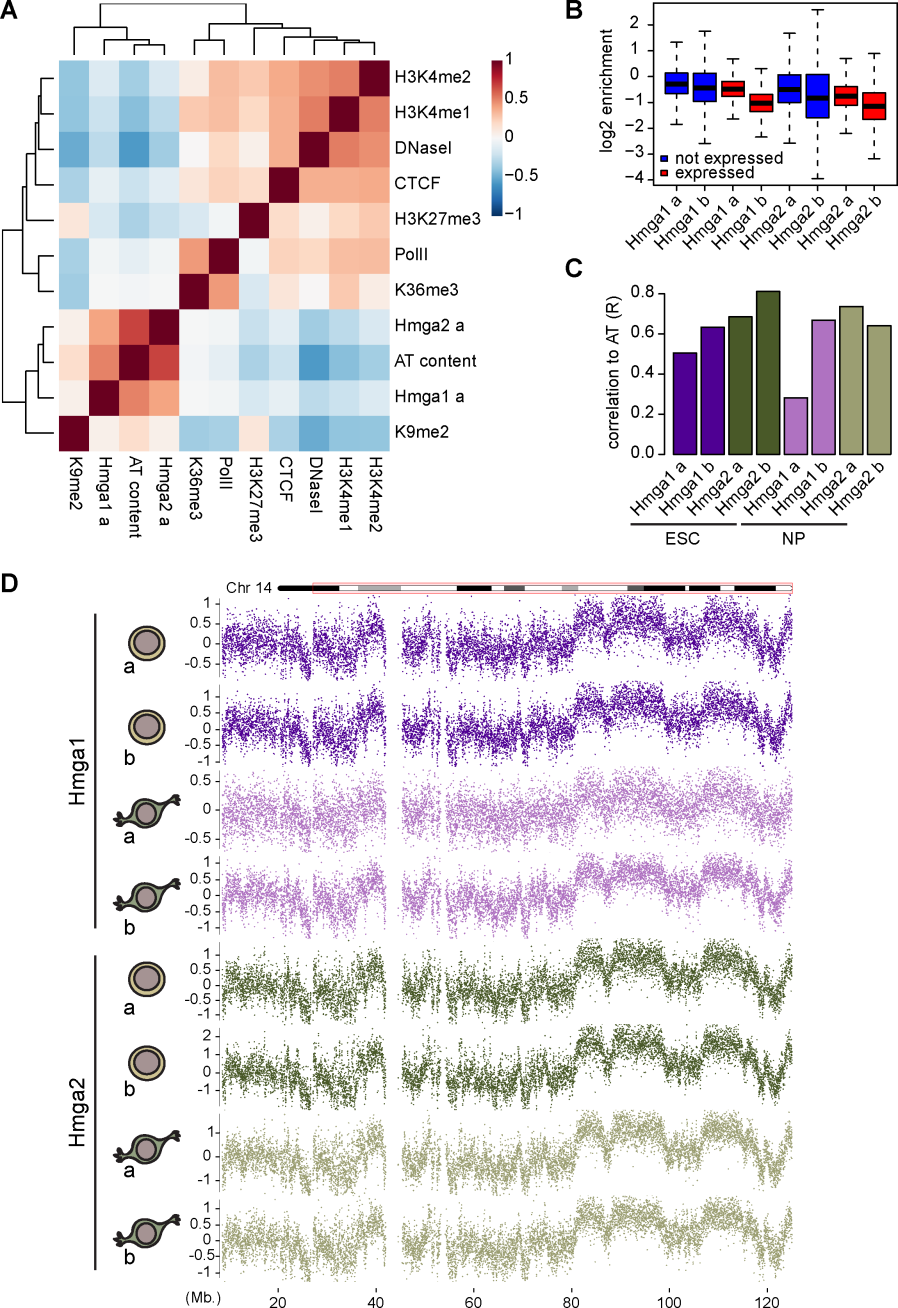
Invariance of Hmga1-2 binding in different chromatin environments and in neuronal progenitor cells. (A) Correlation heatmap of a representative Hmga1-2 replicate versus chromatin marks and AT content. Hmga1 and Hmga2 form a separate cluster with AT content and are weakly anti-correlated with openness (as assessed by DNaseI). Colours indicate the Pearson correlation coefficient. (B) Boxplots showing the distribution of Hmga1 and Hmga2 signal (log2 enrichments normalized over the DBD-mutant) for the indicated ESC replicates over promoters, separated by promoter activity (see Materials and Methods). (C) Barplot showing genome-wide correlations for Hmga1 and Hmga2 replicates in ESC and NP (log2 enrichments over DBD-mutant) with genomic AT content. (D) Log2 enrichments over DBD-mutant for the indicated samples along chromosome 14. Each data-point corresponds to a 10kb tiling window. For better readability, top and bottom 1% of data range are not shown. NP profiles appear very similar to ES-derived profiles (also see S5F Fig).

Local differences in chromatin, through DNA methylation, nucleosome compaction or histone modifications, can modulate the readout of a genomic sequence [42–44]. We therefore investigated the relation of Hmga1-2 to DNA and chromatin features other than AT content. From the results of the PCA (S1H Fig) one might expect low correlations for any such features. Indeed, by contrasting binding to chromatin marks and factors, no relevant correlation manifests itself genome-wide, except for an anti-correlation with euchromatic marks (Fig 3A). This dependence however is of small magnitude within all replicates (S5A and S5B Fig; for a summary plot including all Hmga1-2 samples, see S5C Fig). To further dissect the nature of this anticorrelation, we focused on specific regions in the genome known to undergo extensive chromatin remodeling. Many euchromatic histone modifications are set in an activity-dependent manner, in particular within the promoters of transcribed regions [45,46]. We therefore focused next on promoters and divided them based on activity level (see Material and methods). This revealed no major differences in the levels of Hmga1-2 binding between active and inactive promoters (Fig 3B). Since promoter regions differ largely in their sequence composition, we explored how Hmga binding relates to chromatin in the context of local sequence by further stratifying promoters into CpG islands and CpG-poor promoters. However, in both cases, we again did not observe a major difference in Hmga binding between active and inactive promoters (S5D Fig). Notably, enrichments at CpG island promoters were lower than at non-CpG island promoters in line with the fact that the latter are richer in AT [47].

The experiments discussed so far highlight that Hmga1-2 proteins bind to DNA in a DBD-dependent manner and that binding correlates genome-wide with AT-richness but not with specific chromatin marks. In order to test if this behavior is not limited to stem cells and to test differential chromatin recruitment, we collected Hmga binding data in a different cell type. The utilized stem cells can be readily differentiated to neuronal progenitors (NP) for which we and others have already generated a variety of epigenomic maps [48–51]. In the used differentiation paradigm, not only do these cells change function, identity and transcriptome, but they also become post-mitotic [52]. Furthermore differentiation entails loss of pluripotency, which has been argued to be characterized by unique global chromatin changes [53]. Notably Hmga2 is endogenously expressed in neuronal progenitor cells providing another rationale to monitor Hmga1-2 binding in these cells. Upon neuronal differentiation we performed bioChIP followed by sequencing for both proteins and calculated enrichments over DBD-mutants at genomic tiling windows. As in the case of ES cells, hierarchical clustering results in a cluster grouping WT samples with AT content and a second cluster consisting of all DBD-mutants (S5E Fig).

Indeed, similarity in binding as compared to ESC can be appreciated visually by inspecting the binding pattern along an entire chromosome (Fig 3D). The absence of reproducible differences between ESC and NP replicates extends this observation and argues against a consistent role for chromatin in modulating DNA binding (S5F Fig). The good correlation values between samples and AT content confirm similar binding preferences at the majority of sites (Fig 3C and S5G Fig). Thus the dynamic and well-documented changes in chromatin that occur during loss of pluripotency, gain of neuronal identity and exit from the cell cycle [49,54,55] show limited effect on genomic location of Hmga proteins, which remains effectively a function of DNA sequence.

### Hmga protein binding coincides with broad and stable chromosomal features

**Fig 4:**
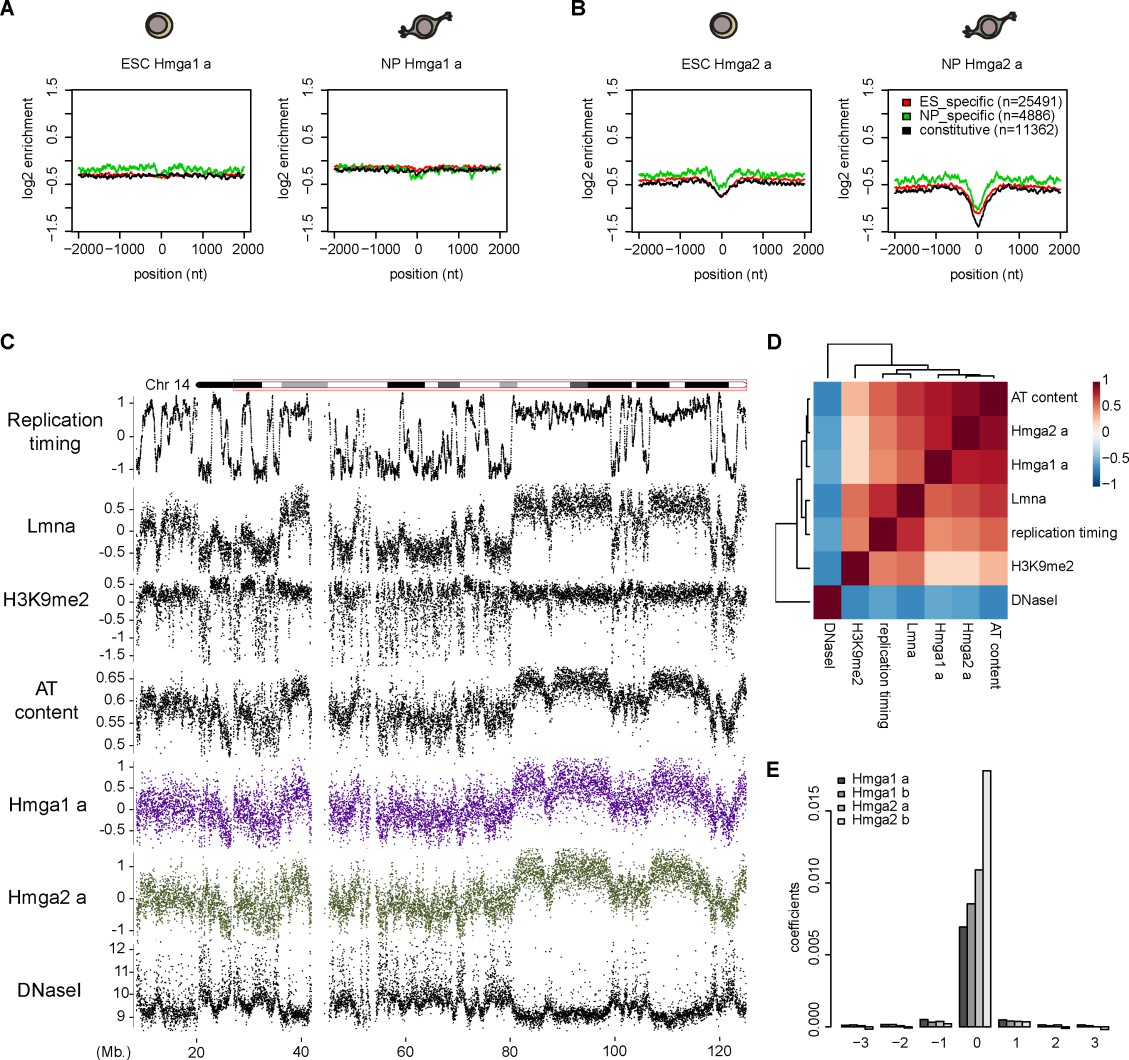
Genomic distribution of Hmga-enriched regions and AT-rich DNA. (A) Average profiles of log2 enrichment values over DBD-mutant at LMR regulatory regions. Shown are ESC and NP signal for replicate “a” of Hmga1. Average signal (smoothed over 51 nts) is shown over a 4 kb window centered at ESC-, NP-specific and constitutive LMR midpoints, shown in red, green and black respectively. This reveals lack of Hmga1 enrichment at both constitutive and cell-type specific regulatory regions. (B) Same as in (A) for replicate “a” of Hmga2. A depletion rather than an enrichment is observed at the indicated regulatory regions. (C) Chromosome-wide profiles of the indicated genomic and epigenomic features in ESC. Each datapoint represents the signal over a 10kb tiling window (replication timing = mean late/early S-phase ratios, Lmna = DamID LaminA, Hmga = input-normalized enrichment over DBDmutant, H3K9me2 = H3K9me2 enrichment over input). (D) Genome-wide correlation heatmap of replication timing, LaminA, H3K9me2, DNaseI cut frequency, AT content and Hmga1-2 as in Fig 1C (10kb tiling windows, colors indicate the Pearson correlation coefficient). (E) Linear model of Hmga protein binding at 1 kb windows based on the AT content of the window itself and the 3 neighboring ones, both upstream and downstream. Plotted are the values of the coefficients for each spatial position grouped by sample. The AT content of the window itself has by far the largest coefficients and contributions from neighbouring windows lead to negligible improvements in predictive power (cf S7E Fig).

Having established that binding of Hmga1-2 is not influenced by sites of open chromatin or presence of local histone marks, we set out to characterize in more detail Hmga1-2 targets in the genome. First, we specifically profiled binding over distal regulatory regions since it was previously reported that Hmga proteins bind subsets of these [20]. Fig 4A and 4B illustrate enrichment values for Hmga1-2 at regions of low DNA methylation (LMRs) in ESC and NP, which we have previously shown to represent distal regulatory regions [48]. We do not observe any enrichment in binding of Hmga1 nor Hmga2 at these sites (Fig 4A and 4B). At LMR centers, a different profile for Hmga1 as compared to Hmga2 can be observed (Fig 4A and 4B). However, this does not seem to be a robust feature as the difference is absent in a second replicate (S6A Fig). This lack of binding is not a feature of enhancer definition or activity. If we use other chromatin features that mark either primed (only H3K4 monomethylation) or active enhancers (both H3K27 acetylation and H3K4 monomethylation) [56] we observe a similar lack of binding (S6B and S6C Fig).

Since Hmga1-2 binding seems largely invariant between cell types and at low resolution appears to cover broad regions defined by high AT content, we next asked whether enriched regions coincide with broad chromosomal features that are known to be largely invariant. These include constitutive heterochromatin, which is characterized by low histone acetylation, high H3K9me2 and high cytosine methylation [57]. Additionally, these regions tend to replicate late during the S-phase of the cell cycle [58]. Importantly with respect to our work, constitutive heterochromatic regions show higher than average AT content [59], due to high prevalence of major and minor satellite repeats and transposon integration events [60]. Due to their large size, such regions are best observed at the chromosomal scale. We accordingly determined Hmga1-2 enrichments over 10 kb windows when comparing them to hallmarks of heterochromatin (Fig 4C).

While Hmga1-2 binding was only weakly correlated to H3K9me2, it displayed good correlations with replication timing [61] and the presence of LaminA [62], with LaminA showing the highest correlation (Fig 4D). LaminA locates to the inner nuclear membrane and is a well-known nuclear organizer of heterochromatin [63]. Importantly, even though in all replicates Hmga binding is well correlated to heterochromatic marks such as LaminA, it clearly displays the largest correlation to AT content (S7A and S7B Fig), suggesting that sequence composition rather than heterochromatic marks determines Hmga binding.

As Hmga binding appears domain-like at a large scale, we wondered whether binding is simply a function of the particular genomic distribution of AT-rich DNA or whether some form of spreading could play a role. Towards this goal we built a linear model that predicts binding over a 1kb window based not only on the AT content of the window in question, but also its immediately neighbouring windows (Materials and methods). Importantly, the correlation of a window’s AT content to the AT content of its flanking windows is not above 0.6 (S7C and S7D Fig), thus containing sufficient additional potentially predictive information. The model fit results in coefficients that are high only for the central window, suggesting that surrounding windows play little role in explaining Hmga binding in the central segment (Fig 4E). This is confirmed by the lack of an improvement in predictive power compared to a model that only includes the AT content of the central window (S7E Fig).

Given that Hmga proteins have been shown to bind mouse major satellite DNA *in vitro* [64,65] and show strong signal at centromeric heterochromatin in the nucleus (Fig 1C and [66]) we also systematically investigated the binding to different classes of repeats (S8A–S8C Fig). This indeed reveals preferential binding to a subset of repeats. However, this differential binding is mainly a function of AT richness as we observed for the non-repetitive part of the genome. Importantly this analysis confirms the previous observations of Hmga binding to major satellites (S8C Fig).

In summary, these findings reinforce our observation that Hmga proteins bind the genome preferentially at regions of higher AT content, which, as a consequence of genome evolution, tend to overlap with large, heterochromatic domains. As we do not detect a dependence of binding on sequence composition of the surrounding regions at the kilobase scale, Hmga1-2 binding to AT-rich DNA thus appears to be determined locally, whilst the organization of mammalian genomes in isochores [37,38] explains the appearance of broad regions of enrichment at Mb resolution.

### Loss of Hmga1 has limited effect on transcriptional output

Our results thus far reveal that Hmga binding to the genome mainly occurs outside of regulatory regions. This is somewhat in contrast to previous *in vitro* and *in vivo* studies that suggested that Hmga1 functions as a co-activator by stabilizing the pre-initiation complex or the enhanceosome, an enhancer-associated protein complex contacting active promoters [18,19,21]. In an attempt to further test the role of Hmga1 on global transcriptional regulation in ESC, we generated a cell line that lacks Hmga1 protein using CRISPR-based mutagenesis (S9A and S9B Fig). Collaterally, this KO cell line further allowed us to test whether binding of bioHmga is different in the presence or absence of the endogenous protein. To this end, we reintroduced either Hmga1 or Hmga2 proteins in the KO background (S9B and S9C Fig), with Hmga1 protein expression restored to levels comparable to WT, and repeated bioChIP experiments. These experiments revealed a genome-wide distribution that was superimposable to Hmga1 and Hmga2 binding in presence of endogenous Hmga1 (S9D Fig). Accordingly, the correlation with AT content was also captured (S9E and S9F Fig) and no residual difference in binding could be detected with respect to the experiments performed in the WT background (S9G Fig). Together these data argue that observations made in the WT background reflect the genuine binding preference of Hmga proteins.

**Fig 5:**
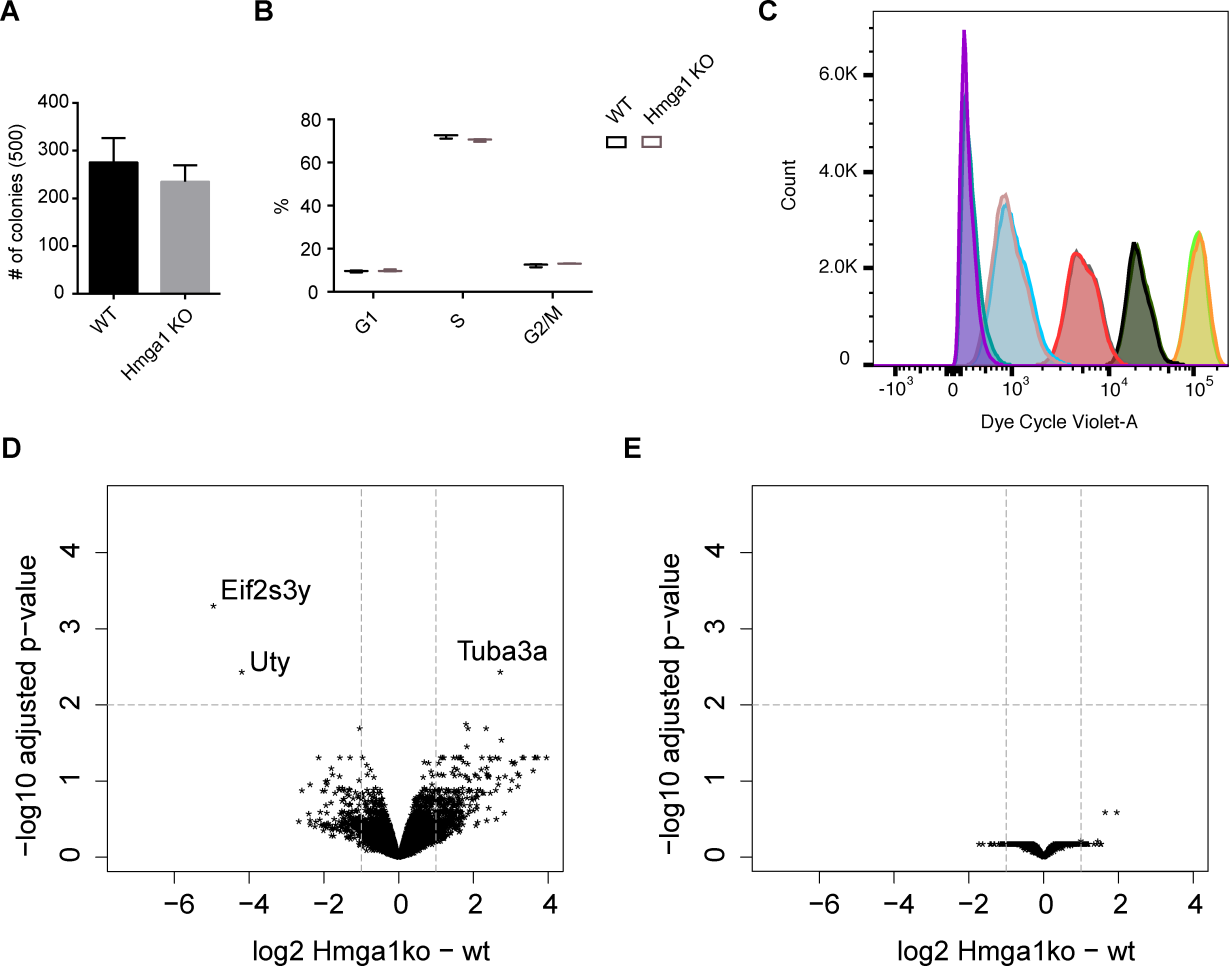
Hmga1 deletion in ESC does not affect transcription globally. (A) Clonogenicity assay of Hmga1 KO and parental cell line. Triplicate biological replicate counts of pluripotent clones out of the indicated number of single cells plated (in brackets). No significant change is observed (one-way ANOVA, CI 95%). (B) Two-way ANOVA analysis of ESC cell cycle distribution data (n = 3) exclude an overall difference between WT and Hmga1 KO samples. Only in S phase a barely significant difference can be seen (adj. p-value = 0.0107). For details, see Materials and methods. Y-axis denotes percentage of cells within a gate. (C) Cell proliferation data of a WT and Hmga1 KO sample during a 4-day time course. The distribution of actively replicating cells in the WT and Hmga1 KO samples are superimposable at any given time point. For colour reference and details refer to S10C Fig and Materials and methods. (D) Transcriptomic comparison of Hmga1 KO vs. parental cell line at the gene level. Gene names are indicated if the gene is significantly differentially expressed (adjusted p-value < 0.01 and absolute fold-change of at least 2). (E) Transcriptomic comparison of Hmga1 KO vs. parental cell line at repetitive regions of the genome as defined by RepeatMasker, excluding repeats lying on the same or opposite strand of annotated transcripts (Materials and methods). Repeat elements show no significant changes (adjusted p-value < 0.01 and absolute fold-change of at least 2). Quantification was performed on the level of RepeatMasker repeat “names”.

Next we determined the global effect of loss of Hmga function. Since Hmga2 is not expressed in mouse ESC this analysis was limited to Hmga1 KO cells, which consequently do not contain any Hmga protein. We observe in ESC a lack of significant changes in colony formation ability and morphology (Fig 5A and S10A Fig), cell cycle distribution (Fig 5B and S10B Fig) and cell proliferation (Fig 5C and S10C Fig). Additionally, we do not observe alterations in the karyotype (S10D Fig). In line with the limited phenotypic differences, global transcriptome analysis of total RNA identifies only 3 genes significantly altered upon loss of Hmga1 (Fig 5D and S11 Fig). Importantly these genes do not include Hmga2 (Fig 5D and S12 Fig), pointing to lack of a shared regulation.

Similar to the absence of transcriptional deregulation at the gene level, we did not observe deregulation of repeat elements (Fig 5E and S14 Fig), which could have been potential targets due to the colocalization of Hmga1-2 enriched regions and heterochromatin. Additionally, ESC lacking Hmga differentiate normally upon neurogenic stimuli (S15A and S15C Fig), and heterochromatin organization does not appear affected at the post-mitotic neuronal stage (S15D Fig), as it is not in stem cells (S1G Fig).

Given the lack of detectable protein products in the Hmga frameshift mutant and matching changes in RNA abundance and structure (S13A–S13C Fig), it seemed unlikely that the lack of transcriptional response could be due to traces of aberrant Hmga1 protein. Nevertheless, we additionally generated a cell line where the entire Hmga1 gene is deleted (S16 Fig). Similarly, this line shows almost no transcriptional changes (S17 and S18 Figs). In summary, it appears that the very limited transcriptional effects upon loss of Hmga function are compatible with our protein location data (preferential binding outside of regulatory and gene-dense regions) and provide no clear evidence for a role for Hmga1 in influencing the output of a particular set of genes.

## Discussion

In order to shed light on the *in vivo* binding preferences of Hmga1 and Hmga2, we applied RAMBiO [32] to mouse ESC and NPs. This approach enabled a rigorous and genome-wide assessment of the preferred *in vivo* DNA and chromatin substrate of Hmga proteins in both cell types.

The observed absence of focal binding of Hmga proteins is backed up by several controls and normalization steps to account for potential contributions of biases that are frequently observed in genome-wide ChIP experiments [29,36]. This exemplifies an inherent problem of ChIP of chromatin components or DNA-binding proteins with low complexity motifs that might bind to any open region in chromatin as compared to transcription factors that bind to more complex and thus less frequently occurring motifs. Here we first performed input normalization, and assessed the impact of mutations in key residues of the DBDs in order to be able to account for such biases.

PCA analysis showed that a single principal component was able to explain almost half of the total variance and this first principal component was strongly correlated to AT content. Visualization of the actual data highlighted reproducible binding between replicates and loss of this binding upon mutation of the DNA-binding domain. These controls were necessary to convincingly expose that the genome-wide distribution of Hmga1-2 is a direct function of DBD affinity for DNA. The dependence on AT content was further confirmed by a direct comparison of Hmga1-2 enrichments to AT content. For both proteins the majority of the genome contains a sufficient density of A or T nucleotides to elicit a response in terms of binding. However, the binding to a highly abundant sequence feature directly explains the overall lack of focal binding and thus precludes the use of algorithms for peak detection. This is highly reminiscent of our previous experience with proteins reading or writing methylated CpGs [31,32]. In turn this required a regional analysis as a function of AT content.

Our findings reveal similar AT-dependence for both proteins and a comparable range of enrichments. However, Hmga1 shows higher noise levels in AT readout, pointing to either lower affinity for DNA or higher sensitivity to chromatin cues. The latter however seems unlikely given the absence of genome-wide correlations with features other than AT content. Together these results argue that genomic binding of Hmga1 and Hmga2 in stem cells is entirely encoded in the respective and highly similar DBDs.

To further evaluate to which extent DNA sequence was the sole determinant of binding, we asked whether a different chromatin environment was able to modulate affinity for AT-rich DNA, either at subsets of regions in the same cell-type or genome-wide in a differentiated post-mitotic cell. In both cases, enrichments were not modulated by the different chromatin states. Importantly, this finding argues that the Hmga1 and Hmga2 binding modality is conserved in other cell-types and cellular states, provided that a functional DBD is expressed. While it seems unlikely, we cannot exclude that Hmga might show varying binding behaviors in other cell types, e.g. due to post-translational modifications [67,68], which might account for the inconsistent observations in the literature. Regardless, our results elucidate the nature of the long-ago proposed preference of Hmga1-2 for AT-rich DNA, namely we show that specific binding occurs throughout the genome over a continuum of affinities with the exception of sites where A and/or T bases are rare such as in CpG islands. Proteins that share a similar dependence on low complexity DNA motifs are proteins of the MBD protein family, the majority of which display a linear relationship between binding and density of methylated CGs [32].

From a biochemical perspective, mechanisms of AT recognition could be read out of base or shape. Indeed the pattern of hydrogen bond donors and acceptors in the minor groove does not allow a discrimination of A:T and T:A nor G:C and C:G base pairs [69]. Thus minor groove binders like Hmga proteins may directly recognize degenerate sequences of the type W_n_ or W-rich (where W stands for A or T nucleotides, IUPAC nomenclature). Alternatively, Hmga proteins may recognize specific DNA shapes. AT-rich sequences are indeed often associated with altered minor groove shapes and in particular A-tract, ApT and ApA (TpT) sequences induce narrowing of the minor groove [70]. In such instances, arginine-mediated recognition of the enhanced negative electrostatic potential offers a mechanism for sequence-specific readout from DNA shape. *In vitro* studies however tend to support the base readout mechanism [71], which is also in line with our observation that including DNA shape results only in a minor improvement over the simple mononucleotide model. Hmga was also suggested to increase IFN-beta enhanceosome assembly through DNA bending [72] even though it is not part of the enhanceosome structure [73]. Given the limits in resolution of our approach and the activity profile of enhancers in ESCs and NPs we cannot discriminate which of these mechanisms is preponderant *in vivo*.

It is known that regions of higher AT content can overlap with heterochromatic DNA. Accordingly, in ESC, regions of higher Hmga1-2 enrichment are broad, replicate late in S-phase and correlate weakly with methylation of lysine 9 of histone H3, all of which are known hallmarks of heterochromatin. Another feature of heterochromatin is binding to the nuclear periphery, which can be assessed through the quantification of the interaction with LaminA, a protein localizing at the inner nuclear membrane. Indeed LaminA displays the second highest correlation with Hmga protein enrichment (after AT content), opening the possibility that Hmga1-2 might be involved in the sub-nuclear localization of heterochromatin since peripheral domains are maintained in a lamin-independent fashion in ESC [62]. It is tempting to speculate that Hmga proteins might function in nuclear organization and that this might only become obvious in terminally differentiated cells and account for the organismal phenotype of loss of Hmga [74]. Furthermore, Hmga might have specific functions in DNA replication, repair or in the organization of the epigenome [4] that we have not tested in detail in our study. While we cannot formally exclude a potential functional compensation by other Hmg proteins, this seems unlikely given that Hmgb and Hmgn proteins are structurally unrelated, with different binding domains and location [75,76].

As heterochromatic regions tend to be gene-poor, Hmga1-2 binding appears to be depleted in regions of high gene density and indeed shows no enrichments at active promoters or distal regulatory regions. This observation is in contradiction to the recently reported enrichment of Hmga2 at gene regulatory regions, albeit observed in a different model system [28]. While we can only speculate about the nature of this difference, we note that Singh et al. did not control for potential biases towards open chromatin by comparing to input chromatin or tested DNA-binding mutants. Regardless, our location data suggests that Hmga proteins are pervasively distributed across the genome and that they do not function as direct regulators of transcription.

In agreement with this model, we observe very limited transcriptional effects when deleting Hmga1 in ESC. Taken together, these results challenge the notion of a central role for the Hmga family of proteins in transcriptional regulation [77]. Instead our findings are more compatible with a recent observation that connects human Hmga1 with genome organization via proper positioning of chromosomal domains [78].

## Materials and methods

### Generation and characterization of transgenic ESC lines and cell culture techniques

The RAMBiO approach has previously been described [32]. Here below is a summary of the relevant procedures adopted in this work.

ESC (159 background, which is a mixed 129Sv-C57Bl/6), were cultivated on feeder cells or 0.2% gelatine coated dishes. ESC growth medium consisted of DMEM (Invitrogen) supplemented with 15% fetal calf serum (Invitrogen), 13 nonessential amino acids (Invitrogen), 1 mM L-glutamine, LIF, and 0.001% beta-mercaptoethanol. Multipotent Pax6-positive radial glial neuronal progenitors were obtained as described previously [49,52].

For construct design cDNAs were amplified from a random hexamer reverse transcription cDNA library (Superscript III, Invitrogen) generated from RNeasy extracted total RNA (QIAGEN, 74104) and sequence verified or alternatively ordered for gene synthesis. Mutations in the DBD of Hmga1-2 where targeted to the core RGR motif of the three AT-hooks. Basic and bulky arginine residues were substituted with small and polar cysteines. Mutations targeting the same residues were previously shown to impair DNA binding in vitro and in vivo [11,79,80]. The amino-acid sequence of the proteins investigated in this study is available in S1 Text.

Constructs were then cloned into pL1-CAG-bio-MCS-polyA-1L. The two inverted L1 Lox sites allowed CRE-mediated integration into a unique genomic site. Gancyclovir (6 μM) resistant clones were selected and tested for direction of the integration through junction-PCR. The parental cell line expresses BirA-V5 biotin ligase under the CAG-promoter, which leads to stable biotinylation of the tagged protein throughout differentiation [32].

Protein expression was assessed by transcriptomics (reported in Fig 1B are read counts per kilobase and million mapped reads obtained from cufflinks v2.0.2 [81] output of Tophat [82] aligned (standard parameters, against Mus_musculus.NCBIM37.67.gtf) data from GSM687305 and unpublished neuronal progenitors RNA-seq data) and western blotting (WB) on whole cell extracts (TNN extraction buffer: 50nM Tris pH 7.5, 250mM NaCl, 0.2mM Na3VO4 0.5% NP-40, 1mM dithiothreitol and protease inhibitors) blotting with specific antibodies (see below) or Streptavidin-HRP (Pierce).

For visualizing protein subcellular localization, cell suspensions were placed on poly-L-lysine for 10 minutes, fixed for 10 min in 3% PFA and permeabilized in 0.1% NaCitrate and 0.1% Triton X-100. After 30 min blocking with 0.1% Tween20, 3% BSA (w/v) and 10% normal goat serum in PBS, detection was performed with Streptavidin-AF568 (ThermoFisher) or primary antibodies over night at 4°C using a Z1 (Zeiss) epifluorescence microscope.

Nuclear-enriched cell preparations were obtained as follows: cell pellets from 1 confluent 10 cm plate were washed twice in cold PBS, lysed in nuclear extraction buffer (n.e.b.) A (20 mM HEPES KOH pH7.5, 10 mM KCl, 1 mM EDTA, 0.2% NP40, 10% glycerol), washed once with n.e.b. A, homogenized 10X with Dounce homogenizer, resuspended in n.e.b. B (20 mM HEPES KOH pH7.5, 10 mM KCl, 1 mM EDTA, 350 mM NaCl, 20% glycerol), homogenized 10X with Dounce homogenizer and cleared supernatant was saved for analysis. All buffers were cooled to 4°C and supplemented with 1mM dithiothreitol and protease inhibitors before use.

Primary antibodies used were Lamin B1 Santa Cruz (C-20) or Abcam (ab16048), Hmga1 Active Motif (39615) and Hmga2 R&D Systems (AF3184).

BD Pharmingen BrdU FITC Flow Kit (BD Biosciences) was used for cell-cycle profiling, whereas CellTrace Violet Cell Proliferation Kit (Thermo Fisher) was used for assaying cell proliferation capability. In both cases, manufacturers’ protocols were followed and data was acquired with a LSR II Flow Cytometer (BD Biosciences).

### CRISPR design and KO strategy

KO strategy relied on introducing frame-shift mutations in the coding sequence of Hmga1 via CRISPR-Cas9 induced indels. We targeted an intron-exon junction in the Hmga1 gene in order to avoid off-targets caused by the presence of pseudogenes [83].

Tools used for CRISPR-Cas9 guide design were http://crispr.mit.edu and http://www.e-crisp.org/E-CRISP/, which led to the selection of the guide GTCCCCTAGGAGGCTCACCC. A pX330 plasmid expressing CRISPR-Cas9 and guide RNA together with a reporter expressing Puromycin-2A-mCherry were co-transfected in ESC. On the following day, Puromycin (2 μg/mL) was added and cells were kept under selective media overnight. Media was refreshed the next day and after single cell plating, clones were isolated. After PCR amplification of a 700 bp region centered on the CRISPR guide, indels were analyzed by Sanger sequencing. WB with a specific antibody was performed to confirm absence of the targeted protein.

For the full-deletion cell line, the same approach was used as for obtaining the KO cells, with the simultaneous addition of GTGAGTCTGGGGGAGATGCA (5’ UTR) and GAAGTTAGCCTTGTCAGGAT (3’ UTR) sgRNAs.

Primers used for the screening PCRs described in S16 Fig were: internal (CTTGAGTGACAGTTCTCCCCAGG and GGGCCAGGGGTTAAAACATAAGG), external (AAGTGGGTGGAGCCAACATC and TGCCCTTGCCCTAAGGTAG) and control region (*Hmgb1* locus, GTGTTCTCCTTACTATATGAC and GTAGTGATATACTGTGCAAAG).

### ChIP-seq

BioChIP experiments were performed as described [32] except that after crosslink reversal DNA was purified with QIAquick PCR Purification Kit (QIAGEN, #28104). Briefly cells were fixed for 10 minutes with 1% Formaldehyde at room temperature and incubated for 10 min on ice in the presence of 125mM Glycine. Cells were harvested and treated for 10 min with 10mM EDTA, 10mM TRIS, 0.5mM EGTA and 0.25% Triton X-100 and 10 min in 1mM EDTA, 10mM TRIS, 0.5mM EGTA and 200mM NaCl with subsequent nuclear lysis in 50mM HEPES, 1mM EDTA, 1% Triton X-100, 0.1% deoxycholate, 0.1% SDS and 150 mM NaCl. DNA was purified with Qiagen columns for PCR Purification. Crosslinked chromatin was subjected to sonication in a Bioruptor instrument (Diagenode). ProteinA (Invitrogen) pre-cleared chromatin was either saved as input or incubated with blocked (1%CFSG, 100ng tRNA) Streptavidin-M280 (Invitrogen) magnetic beads over night at 4°C. Beads were washed and treated with RNaseA for 30 min at 37°C, Proteinase K for 3 hours at 55°C, then de-crosslinked over night at 65°C.

For replicate C of Hmga1, cross-linked cell pellets were resuspended in 50 mM Hepes-KOH (pH 7.5), 140 mM NaCl, 1 mM EDTA, 10% Glycerol, 0.5% NP-40, 0.25% Triton X-100 for 10 min on ice (membrane lysis). Nuclei were collected by centrifugation and resuspended in 10 mM Tris-HCl (pH 8.0), 1 mM EDTA, 200 mM NaCl, 0.5 mM EGTA for 10 min RT (removal of detergents). Nuclei were collected by centrifugation and resuspended in 10 mM Tris-HCl (pH 8.0), 1 mM EDTA, 0.1% Deoxycholate, 200 mM NaCl, 0.25% N-Lauroylsarcosin, 0.5 mM EGTA.

Crosslinked chromatin was subjected to sonication in a Bioruptor instrument (Diagenode). Triton X-100 to 1% final concentration was added before SAV-IP. Subsequent steps were performed as for the other replicates.

Libraries of extracted DNA from the IP and input (50 μl of IP) fraction were prepared according to the manufacturer’s protocol using either the NEBNext ChIP-Seq Library Prep Master Mix Set for Illumina (New England BioLabs, #E6240) or the NEBNext Ultra DNA Library Preparation Kit (New England Biolabs, #E7370L).

DNA was measured using NanoDrop 3300 Fluorospectrometer (Witec AG) and Qubit dsDNA HS Assay Kit (ThermoFisher). Size-selection was performed using Agencourt AMPure XP beads (Beckman Coulter, # A63880) before PCR amplification with NEBNext Multiplex Oligos for Illumina (New England BioLabs, #E7335). PCR amplification was performed for 6 to 12 cycles using indexed primer and cycling conditions according to Illumina recommendations. Adapter-ligated and amplified DNA was purified using AMPure XP beads. Before pooling, the size distribution was checked on an Agilent Bioanalyzer 2100 using Agilent High Sensitivity DNA kit (Agilent technologies, #5067–4626).

### RNA-seq

For RNA-seq, two micrograms of total RNA was used from at least two independent cultures harvested on different days. RNA was isolated with the RNeasy mini kit (Qiagen) with on-column DNA digestion and ribosomal RNA was depleted using the Ribo-Zero rRNA removal kit (Epicentre). Strand-specific RNA-seq libraries were prepared from rRNA-depleted samples using the ScriptSeq v2 protocol (Epicentre) following producer’s instructions. Up to 7 samples with different barcodes were mixed at equimolar ratios per pool. Sequencing was performed on an Illumina HiSeq 2500 machine (50 bp read length, single-end, according to Illumina standards).

### Data analysis

#### Annotations

Promoters were defined using the UCSC knownGene annotation via the R package TxDb.Mmusculus.UCSC.mm9.knownGene (Marc Carlson and Bioconductor Package Maintainer, TxDb.Mmusculus.UCSC.mm9.knownGene: Annotation package for TxDb object(s), R package version 3.2.2., 2015). For each transcript, promoters were defined as +/-1000 nucleotides around transcription start sites and only promoters that were at least 80% mappable were retained (see section ChIP-seq for details). For each gene, the promoter with highest levels of PolII ChIP-seq enrichment over input was selected (see section ChIP-seq for details). For this initial annotation, ChIP-seq and input reads were shifted by 60nts, assuming a fragment size of 120nts. Promoters on chrM and random chromosomes (chr*_random) were removed and only promoters that did not overlap with any other promoter were retained for the downstream analysis. RepeatMasker (Smit, AFA, Hubley, R & Green, P. *RepeatMasker Open-4*.*0*, 2013–2015. http://www.repeatmasker.org) repeat annotation was downloaded from UCSC (https://genome.ucsc.edu). CpG islands were retrieved from UCSC (“cpgIslandExt” table) using rtracklayer [84]. Unmethylated (UMRs) and low-methylated regions (LMRs) were determined using MethylSeekR [85] on ESC and NP whole-genome bisulfite data [48] with parameters m = 50% and n = 3 (smallest *n* such that FDR < 5%) as previously described [85]. LMRs were further subdivided into ESC-specific LMRs (ESC LMRs that do not overlap with any NP UMRs nor LMRs), NP-specific LMRs (NP LMRs that do not overlap with any ESC UMRs nor LMRs) and constitutive LMRs (segments which consist of overlapping LMRs from both cell-types (at least 1 ESC LMR and 1 NP LMR), consisting of all the nucleotides of the overlapping LMRs). Enhancer annotation was downloaded from ENCODE [86]. In particular enhancers as defined in [87] were downloaded from (http://chromosome.sdsc.edu/mouse/download/mESC.zip)). For the classification into primed and active enhancers, broad peaks for H3K27ac from [88] were downloaded from GEO (https://www.ncbi.nlm.nih.gov/geo/, accession GSM1000099).

#### ChIP-seq

ChIP-seq and input samples were mapped to the mm9 assembly of the mouse genome using the R package QuasR [89], which internally uses bowtie [90]. Bowtie was run with QuasR default parameters, allowing only for uniquely mapping reads. In all analyses on regions (promoters, windows and repeats), only regions in which at least 80% of all possible overlapping 50-mers were mappable using these alignment parameters were used.

For all samples, fragment lengths were estimated by calculating average profiles over promoters separately for reads mapping to the same strand and to the opposite strand of the respective promoters (function *qProfile* in QuasR with argument “orientation” set to “same” and “opposite”, respectively) and finding the shift that minimizes the root-mean-square deviation between the two profiles.

The number of alignments in promoters, repeats and tiling windows (1kb or 10kb) along the genome were counted using the QuasR function *qCount* using the corresponding estimated shifts (i.e. half the estimated fragment lengths). Enrichments over input were determined as *log2(ns*_*IP*_ *+ 8)–log2(ns*_*input*_ *+ 8)*, where *ns*_*IP*_ *and ns*_*input*_ are the library-size normalized counts of the IP and corresponding input samples in a given promoter, window or repeat. The library-size normalized counts were determined as *ns*_*IP*_ *= min(N*_*IP*,_ *N*_*input*_)*(n_*IP*_*/N*_*IP*_*)* and *ns*_*input*_ *= min(N*_*IP*,_ *N*_*input*_)*(n_*input*_*/N*_*input*_*)* where *n*_*IP*_ and *n*_*input*_ are the raw counts per promoter, window or repeat and *N*_*IP*_ and *N*_*input*_ are the total number of reads mapping to the genome in the IP and input respectively. The counts were normalized to the smaller library size. The pseudo-count of 8 was used to decrease noise levels at low read counts. Enrichment over the DBD-mutant was defined as the difference in log2 enrichments over input (as defined above) of the wild-type and the corresponding DBD-mutant sample. In all analyses, replicates “a” were paired and replicates “b” were paired (for example, “Hmga1 a” with “Hmga1 DBD-mutant a” and “Hmga1 b” with “Hmga1 DBD-mutant b”, the pairing is arbitrary). Hmga1-2 add-back samples were paired with the respective DBD-mutant samples of the wt background.

#### Average profiles

Average profiles over ESC-specific, NP-specific, constitutive LMRs, enhancers, CpG islands and (TA)n simple repeats were calculated using the QuasR function *qProfile* using the estimated read shifts. Normalization to input and DBD-mutant as performed analogously to the normalization in promoters, windows and repeats (see above) where the counts here correspond to the sum of reads mapping to a window of size 51 (201 in the case of (TA)n simple repeats due to the small number of instances and thus higher noise levels (see below)) centered at each position in the profile, summed over all regions of the respective type.

#### Regional profiles

For LaminA DamID, Dam ratios of the loess and quantile normalized data were downloaded from GEO. For the replication timing data, the wavelet-smoothed signal was downloaded from ENCODE (for accessions, see below). For both datasets, window levels were calculated by averaging the signal for each probe mapping to the respective window. For the replication timing data, the average coverage was 1.65 probes per window and 98.4% of windows were covered by at least 1 probe. For the LaminA data, the average coverage was 8.94 probes and 99.96% of windows were covered by at least one probe.

#### Principal component analysis

Principal component analysis was performed on mean-centered log2 enrichments over input in 1kb tiling windows of the genome using the R function *prcomp*. This resulted in the first principal component (PC) loading being negatively correlated to AT content. Since the signs of the PC loadings are arbitrary and a positive correlation to AT content can be more intuitively appreciated, all principal component loadings and, accordingly, all principal component scores were multiplied by -1.

#### Nucleotide models

To check whether more complex sequence features than simple AT content are predictive of Hmga binding, we modeled Hmga binding as a function of the mono-, di-, tri- and tetranucleotide frequencies of each 1kb tiling window with ridge regressions using the R package *glmnet* [91]. Regularization is necessary in such models due to the high correlation between nucleotide counts. Since repetitive elements can potentially bias nucleotide counts, we only used genomic windows that did not overlap with any annotated repeat from RepeatMasker. We also excluded CpG islands from the analysis as they have a different sequence composition than the rest of the genome. As enrichment values (for each sample) were unevenly distributed with most enrichments around 0, we selected, for each sample separately, a subset of windows with a roughly uniform distribution of enrichment values over the entire range of enrichments (in order not to bias the modelling towards values of enrichments around 0). This was done by dividing the data into 12 bins, consisting of a bin from the minimal value to the 2.5% quantile, 10 equally spaced bins between the 2.5% quantile and the 97.5% quantile and 1 bin from the 97.5% quantile to the maximum value, and randomly sampling the same number n of windows from each bin (n corresponds to the number of windows in the bin with the smallest number of windows). This set was further randomly subdivided into two sets of equal size, a training set to infer model parameters, and a test set to determine the model performance. This procedure resulted in roughly 34’000 windows in each training and test set. Ridge regression parameters were determined on the training set of each sample separately with either mono-, di-, tri- or tetranucleotide frequencies as predictors (without intercepts) using the R library *glmnet* [91]. The function *cv*.*glmnet* was used to determine the regularization parameter λ via 10-fold cross-validation on the training set. More specifically, λ was set to “lambda.1se”, which results in the most regularized model such that the error is within one standard error of the minimum mean cross-validated error [91]. The models were then used to assess the predictive power on the test set, which was measured by both Pearson correlation and root-mean-square deviation. The improvement in performance of the di-, tri- and tetranucleotide models compared to the mononucleotide models was minor in terms of either measure. The entire analysis was repeated on all windows outside of CpG islands (not removing windows overlapping repeats), leading to generally lower predictive power, but the same qualitative results.

#### AT-stretch model

Having established that Hmga binding could be well explained by AT content, we wondered whether predictions could be improved by including information about the clustering of As and Ts along the DNA sequence. To this end, we determined, for the same set of windows as for the nucleotide models, the number of stretches of consecutive Ws (W = A or T) of length 1 to L (where L is the largest length found in any of the windows. L = 20 in this case) and used these as predictors for the ridge regression (with offset term). Model fitting and the evaluation of predictive power was done as in the case of the nucleotide models. The improvement in terms of predictive power was only minor compared to the simple mononucleotide model.

#### DNA-structure model

To evaluate whether the local structure of the DNA has an effect on Hmga binding, we extended the mononucleotide model by incorporating structural information as predicted by the R package DNAshapeR **[92]**. DNAshapeR calculates for each pentamer in a given DNA sequence the minor groove width (MGW), the propeller twist (ProT), the helix twist (HelT) and the DNA roll (Roll), using values derived from all-atom Monte Carlo simulations. The former two correspond to the estimated MGW and ProT at the central nucleotide of the pentamer, whereas the latter two correspond to the estimated HelT and Roll between the second and third as well as the third and fourth nucleotide of the pentamer. For modelling, the two values of HelT or Roll were averaged to end up with 4 numbers for each mononucleotide corresponding to the estimated MGW, ProT, (average) HelT and (average) Roll. For each mononucleotide, each structural feature was binned into 5 equally sized bins, and the counts per bin of each structural feature were used as predictor values for the modelling. This resulted in 5*4 = 20 predictors per nucleotide and thus 80 predictors in total. Model fitting and the evaluation of the predictive power were done as in the case of the nucleotide models. Also in this model, the improvement in terms of predictive power was small compared to the simple mononucleotide model.

#### Proximity model

To estimate the effect of spreading of Hmga binding, we used a linear (ordinary least squares) model that predicts binding (log2 enrichment over DBD-mutant as defined above) in 1 kb windows based on the AT content of the respective windows and their 0–3 neighbouring 1kb windows up- and downstream. We used all windows outside of CpG islands and retained only windows where all 3 windows up- and downstream did not overlap CpG islands and were at least 80% mappable. Windows overlapping repeats could not be removed as this would have too strongly reduced the set of possible windows on which to train the model. As in the nucleotide models, windows were sampled such that the distribution of enrichments of the central window were roughly uniform over the entire range of enrichments and windows were randomly split into training and test sets of equal size (for each sample separately). Performance was assessed using Pearson correlation.

Importantly, the correlation of a window’s AT content to the AT content of its flanking windows is not above 0.6 (S7C and S7D Fig), thus containing sufficient additional potentially predictive information (and making regularization unnecessary). The model fit resulted in coefficients that are high only for the central window, suggesting that the surrounding windows play little role in explaining Hmga binding in the central window (Fig 4E). This is confirmed by the absence of a substantial improvement in predictive power compared to a model that only includes the AT content of the central window, measured using Pearson correlation (S7E Fig) or root-mean-square deviation.

#### ChIP-seq analysis on repetitive elements

Repeat analysis was restricted to the subset of repeats from RepeatMasker that have a minimal length of 300 nts due to the limitations in ChIP-seq resolution and the general abundance of As and Ts in the genome that make it difficult to distinguish binding signal stemming from a short repeat versus its surrounding region. Repeats were further filtered out if the fraction of mappable bases was below 80%. Finally, in order to be able to estimate robust enrichment values, only repeat names with at least 10 instances in the genome (after applying the previous filters) were used. This leads to more than 50 reads on average across all input samples (aggregated over all instances of each repeat type) in every enrichment calculation.

#### Copy-number variation analysis

To determine whether there are any differences in karyotype between KO and WT cell lines, we calculated, for each WT and KO ESC input sample, the number of reads in (at least 80% mappable) 1 Mb tiling windows of the entire genome. The log2 counts were then for each sample separately normalized to the mean across all windows. Since GC biases confound the copy number signal, we then performed, for each sample separately, loess regression of mean-normalized log2 read counts against GC content. The resulting residuals were then averaged per chromosome. All WT and all KO values from the respective samples were averaged to determine mean ratios (KO/WT) per chromosome. Individual WT and KO values were all very similar.

#### RNA-seq

RNA-seq reads were mapped to the mm9 assembly of the mouse genome using QuasR. Bowtie was run with mapping parameters "—trim5 3 -m 100—best—strata" (*qAlign* alignmentParameter set to "—trim5 3 -m 100—best—strata"). For multi-mapping reads, one randomly chosen alignment was used for quantification. The first 3 nucleotides from the 5’ end were trimmed (“—trim5 3”) as they showed higher error rates in an initial round of mapping without trimming. Gene-level counts were determined using the UCSC knownGene table [93] via the Rpackage *TxDb*.*Mmusculus*.*UCSC*.*mm9*.*knownGene*. As a stranded RNA-seq protocol was used, only reads on the same strand as the respective genes were counted (QuasR command *qCount(proj*, *TxDb*.*Mmusculus*.*UCSC*.*mm9*.*knownGene*, *reportLevel = "gene"*, *orientation = "same")*). To determine significantly changing genes, limma-trend as part of the limma R package was used [94,95] as recommended when sequencing library sizes vary by less than 3 fold (see limma user guide on www.bioconductor.org). Only genes that had a mean count of at least 10 reads, averaged over all samples, were used. To account for batch effects (in the KO and corresponding WT samples, not in the full deletion and corresponding WT samples), a batch variable was included in the linear modelling which grouped samples if library preparation had been done on the same day and they had been sequenced on the same flow cell. Genes with an adjusted p-value < 0.01 (Benjamini-Hochberg) and an absolute fold-change of at least 2 were called as significantly changing. For S11B and S11D and S17C Figs, gene counts for each sample were scaled to the smallest total number of gene counts in any sample (library-size normalization) and to the average gene length (length-normalization, gene length was defined as the total number of exonic bases) and log2-transformed after adding a pseudo-count of 8. Genes were defined as expressed if the resulting number was at least 7, which is roughly the level which separates the two modes of the bimodal distribution of expression levels. For S11C and S17E Figs, counts were only library-size normalized and log2 differences were determined after adding a pseudo-count of 8.

Repeat quantification was done on the level of Repeat masker repeat “names” using the *qCount* function of QuasR, counting only reads on the same strand as the annotated repeats (argument orientation = "same") and ignoring all repeats that overlap with an annotated transcript in *TxDb*.*Mmusculus*.*UCSC*.*mm9*.*knownGene* (on the same or opposite strand of the corresponding gene). Repeats mapping to transcripts were excluded as their changes in read counts may be due to expression changes of the corresponding genes. Linear modelling and significance calculations were done as for genes. For S14B and S14D and S17D Figs, library size-normalization was done as in the case of gene expression (see above). For S14B and S14D and S17D Figs, length-normalization was done by determining the counts per kilobase of mappable positions of each repeat “name”. After the respective normalization steps, the counts were log2-transformed after adding a pseudo-count of 8 as in the case of gene expression. For S14C Fig, the log2 count distributions were quantile-normalized using the *normalizeQuantiles* function from the limma package [95]. This was necessary since the shapes of the distributions of log2 read counts were different between different samples.

#### Sashimi plots

**To create Sashimi plots, RNA-seq reads were first realigned using SpliceMap**[96]**, using the** QuasR function *qAlign* with default value for the alignmentParameter argument, maxHits = 100 and spliced = TRUE.

Sashimi plots were generated using the sashimi_plot function [97] as part of the MISO software[98], downloaded from the Python Package Index (https://pypi.python.org/pypi/misopy)). For visualization of Hmga1, Hmga2 and Uty gene models, the pre-processed gene annotation in GFF3 format (mm9 Ensemble genes [99] downloaded from UCSC [100]) was downloaded from http://genes.mit.edu/burgelab/miso/annotations/ucsc_tables/mm9/ensGene.gff3.

#### Single locus tracks

To determine the enrichments for the single locus tracks in S2D–S2F Fig, counts were determined in a running window of 401 nts centered at each position in the profile. For each such running window, counts were then scaled to the mean total number of aligned reads across all samples and the scaled counts of all ESC and NP replicates a and b were pooled (separately for IPs and inputs in the WT background of Hmga1, Hmga2, Hmga1 DBD-mutant and Hmga2 DBD-mutant) to increase coverage. Only replicates a and b were used in order to have the same number of samples for each protein and thus comparable coverage. Enrichments over the DBD-mutants were calculated as described in the section ChIP-seq.

All data analysis was performed in R [101], using many packages available at Bioconductor (www.bioconductor.org, [102]). The most used packages not already referenced above were Genomic Ranges for data handling and overlap calculations [103], Gviz for visualization of genomic data [104], the package NMF for creating heatmaps [105] and maptools for point labelling (Roger Bivand and Nicholas Lewin-Koh, 2017. *maptools*: *Tools for Reading and Handling Spatial Objects*. R package version 0.9–2. https://CRAN.R-project.org/package=maptools)).

Data sets generated for this study are available from GEO (https://www.ncbi.nlm.nih.gov/geo/) under accession GSE100407.

Additional ESC data used in this study was downloaded from https://www.encodeproject.org/files/ENCFF001JUP/ and https://www.encodeproject.org/files/ENCFF001JUQ/ (Replication Timing) or from GEO with the following accession numbers: GSM1531435, GSM1531436 (LaminA); GSM1314605, GSM1314606, GSM1543602, GSM1543603 (H3K9me2); GSM632032, GSM632033, GSM632034 (H3K27me3); GSM1000099 (H3K27ac); GSM747542 (H3K4me1); GSM632035 (H3K4me2); GSM747534, GSM747535, GSM747536 (CTCF); GSM747547, GSM747548 (RNA PolII); GSM671103 (input DNA); GSM1657364, GSM1657365 (DNaseI); GSM748786, GSM748787 (DNA methylation).

Additional NP data used in this study was downloaded from GEO with the following accession numbers: GSM748788, GSM748789 (DNA methylation).

## Supporting information

S1 Text Protein sequences. (DOCX)

S1 Fig Features of Hmga1 and Hmga2 expressing cells. (A) Protein alignment between Hmga1 (top row) and Hmga2 (bottom row) obtained with Uniprot through Clustal Omega [106]. Highlighted in grey are the DNA-binding domains, which show notable levels of conservation as assessed by the number of identical (*), highly similar (:) and similar (.) amino acids. The conserved regions where bulky and positively charged Arg residues were mutated to Cys are shown in red.
(B) WB with anti-Hmga2 Ab of whole cell lysate from cells differentiated to the neuronal progenitor stage. A band is visible at the expected height for Hmga2 (for comparison with bioHmga2 see S1F Fig).
(C) WB with anti-SAV conjugated HRP of whole-cell lysate from parental cell line and cells expressing Hmga1 and Hmga2. Although slightly different, expression levels are comparable between both proteins.
(D) Clonogenicity assay for the indicated cell lines showing similar pluripotent potential for cell lines expressing the biotinylated constructs. Displayed are the mean and standard deviation of 3 replicate counts of alkaline phosphatase positive colonies 5 days after plating to clonal density (number of single cells in brackets). None of the engineered lines show a significant difference to the parental cell line (one-way ANOVA, Bonferroni’s multiple comparison correction, CI 95%).
(E) WB with anti-SAV conjugated HRP of whole cell lysate from parental cell line and cells expressing Hmga1 or mutated Hmga1. In the last lane, a lower molecular-weight band (due to a smaller mass of the side chains of the cysteine residues as compared to the WT arginines) representing mutated bioHmga1 is visible and shows a comparable expression level to bioHmga1. Blotting with SAV was chosen because the mutated bioHmga1 protein runs at the same position as the untagged Hmga1.
(F) WB with anti Hmga2 Ab of whole-cell lysate from parental cell line and cells expressing Hmga2 or mutated Hmga2. As expected from the lack of mRNA signal (Fig 1B), Hmga2 is not expressed in ESC. A lower molecular weight band representing mutated bioHmga2 is visible and shows a comparable expression level to bioHmga2.
(G) Co-localization analysis of bioHmga1 and Hmga1 by IF: SAV-conjugated fluorophore (pseudo-coloured red) and secondary anti-rabbit (coloured green). DAPI was used to stain the cell nuclei (coloured blue). Top-left, Hmga1 KO sample (see Fig 5 and S9 and S10 Figs for further characterization); top-middle, WT parental cell line; top-right, bioHmga1-expressing cell line. Bottom, scatterplots of pixel intensities (y-axis, red channel; x-axis, green channel) and Pearson’s correlations, as determined by the “Colocalization Threshold” plugin for ImageJ 1.51n (https://imagej.nih.gov/ij/), for bioHmga1 (left) and bioHmga2 (right) expressing cell lines. High levels of spatial correlations are detected in both instances.
(H) Correlation heatmap of the PC loadings, various chromatin marks as well as AT content. While the PC1 loading is strongly associated with AT content, all other PC loadings show no or only weak correlations with the investigated features.
(TIF)

S2 Fig Hmga binding to representative elements and region. (A) Barplot showing genome-wide correlations for all Hmga1 and Hmga2 replicates in ESC and NPs (log2 enrichments over input) with respect to genomic AT content.
(B) Log2 enrichments over input for the depicted samples on chromosome 14. Each dot represents the log2 enrichment of IP over input in a window of size 10kb. Gaps indicate regions with low mappabilty (below 80%). Top and bottom 1% of data range are not shown to enhance readability. Data for replicate c of Hmga1 and Hmga1 DBD-mutant was obtained using a different ChIP protocol and highlights the robustness of the results (see Materials and methods).
(C) Hmga binding is depleted at CpG islands as it follows AT content. Average profiles were calculated relative to CpG island starts (nt). Enrichment denotes enrichment over the corresponding DBD-mutant. AT content is shown in grey (dashed line). AT content is not shown directly around CpG island starts (position 0) since the start position is by definition a CpG, resulting in an artificial local dip in AT content. All values denote a running mean over 51 nucleotides.
(D)-(E)-(F) Single locus tracks of log2 enrichments over the respective DBD-mutants at three (TA)n simple repeats with strong binding. To increase coverage, the counts of all Hmga1 samples, all Hmga2 samples, all Hmga1 DBD-mutant samples and all Hmga2 DBD-mutant samples were separately pooled and aggregated in a running window of 401 nts before determining enrichments (see Materials and methods). The locations of the (TA)n repeats are marked by arrows.
(TIF)

S3 Fig Nucleotide models to explain binding. (A) Inferred ridge regression coefficients are internally consistent. Shown are scatter plots of the inferred coefficients for each mono-, di-, tri- or tetranucleotide versus the coefficients of its reverse complements for the respective models. As the ChIP-seq data does not contain any strand information, the inferred coefficient for a nucleotide should be very similar to the coefficient of its reverse complement (the nucleotide frequencies that act as predictors for the ridge regression are determined from the plus strand sequence only). This is indeed the case as evidenced by the high Pearson correlation coefficients (R).
(B)-(C) Inferred ridge regression coefficients for the mono- (left) and dinucleotides (right) for each sample. For Hmga WT proteins high coefficients are apparent for A and T nucleotides and combinations thereof. The “CG” dinucleotide coefficients are high in all samples which may reflect the unspecific recruitment to accessible regions, which tend to be CpG- and GC-rich [47].
(D)-(E) Inferred ridge regression coefficients for tri- (left) and tetranucleotides (right) consisting of only As and/or Ts. The inferred contributions of the different tri- or tetranucleotides are highly comparable. Nonetheless, there is a subtle, but reproducible preference of polyA or polyT stretches over polyAT or polyTA stretches. However, this and other subtle differences are too small to substantially improve the predictive power of the tri- or tetranucleotide model over the simple mononucleotide model (Fig 2C).
(TIF)

S4 Fig Binding as a function of AT-stretch length and DNA shape. (A) The improvement in predictive power of the AT-stretch model over the simple mononucleotide model is small. R stands for the Pearson correlation coefficient.
(B) Inferred coefficients of AT-stretches of length L scale linearly with L. This is in agreement with a model where the addition of one extra A or T leads to a constant increase in binding, which is in agreement with the predictions of the simple mononucleotide model.
(C) The improvement in predictive power of a model that incorporates local structural features of the DNA (see Materials and methods) in addition to mononucleotide frequencies is also only minor. R stands for the Pearson correlation coefficient.
(TIF)

S5 Fig Hmga binding relative to chromatin marks, expression and differentiation state. (A) Scatterplots of log2 enrichments over DBD-mutant versus chromatin features and AT content for a representative Hmga1 replicate (1kb tiling windows, R: Pearson correlation coefficient).
(B) Same as in (A) for a representative Hmga2 replicate.
(C) Correlation heatmap of cut frequency of DNaseI, AT content, chromatin marks, CTCF and bioChIP samples (log2 enrichments over DBD-mutant, 1kb tiling windows, colours indicate the Pearson correlation coefficient). Hmga1-2 correlate most strongly with AT content and form a separate cluster, in agreement with the PCA.
(D) Distribution of DBD-mutant-normalized log2 enrichments for the indicated samples over promoters stratified into different categories: promoters overlapping (CGI) or not overlapping a CpG island (not CGI), expressed or not expressed. Hmga1 and Hmga2 enrichments are generally lower at CpG island promoters. Non-expressed non-CpG island promoters (blue boxplots) appear to have slightly larger enrichments than expressed non-CpG island promoters (in red).
(E) Correlation heatmap of log2 enrichments over input for all NP samples, showing that as in ESC, WT and DBD-mutant samples form separate clusters and WT samples show good correlations to AT content.
(F) Scatterplot and Pearson’s correlation of the log2 difference between NP and ESC enrichment values (over DBD-mutant) for two replicates of Hmga1 (left) and Hmga2 (right) over 1kb tiling windows indicating that there are no reproducible differences in binding between ESC and NPs.
(G) Scatterplot and Pearson’s correlation of Hmga1 and Hmga2 samples versus AT content in ESC (top row) and NPs (bottom row), illustrating the nature of the positive correlation for all samples (1kb windows, log2 enrichments over DBD-mutant).
(TIF)

S6 Fig Hmga binding to enhancer regions. (A) Average profiles at LMRs [48] of log2 enrichments over DBD-mutant for two replicates of Hmga1 (top row) and Hmga2 (bottom row) in ESC and NP. Values were smoothed over 51 nts.
(B) Average profiles at primed enhancers (marked by K4me1 and not marked by K27ac (K4me1+K27ac-)) and active (marked by both K4me1 and K27ac (K4me1+K27ac+)) enhancers for two Hmga1 replicates in ESC. Enrichments denote enrichment over the respective DBD-mutants.
(C) Same as in (B) for two Hmga2 replicates in ESC.
(TIFF)

S7 Fig Correlation between Hmga binding and heterochromatic features. (A) Scatterplot of log2 enrichment values over DBD-mutant in 10kb tiling windows for one representative Hmga1 (top) and Hmga2 (bottom) replicate versus the indicated heterochromatic features, AT content or DNaseI cut frequency (same data as in Fig 4C). R denotes the Pearson correlation coefficient.
(B) Genome-wide correlation heatmap of all measures in (A) and all ESC Hmga1 and Hmga2 replicates (10kb tiling windows, colours indicate the Pearson correlation coefficient).
(C) Correlation heatmap of AT content in the set of neighbouring 1kb windows used for linear modelling (see Materials and methods). 0 refers to the central window, +/- *n* to the *n*-th neighbouring window downstream or upstream, respectively. The correlation between neighbouring windows is not very strong, thus containing potential additional information for linear modelling.
(D) Example scatterplot of AT content in the central versus the directly neighbouring downstream window (+1). R indicates Pearson correlation coefficient.
(E) Pearson correlation of predicted log2 Hmga enrichments (over DBD-mutant) versus measured values for linear models that use only the AT content of the window itself or the AT content of the window itself as well as of 1–3 neighbouring windows both up- and downstream. There is no substantial increase in predictive power when the AT content of neighbouring windows is taken into account.
(TIFF)

S8 Fig Hmga binding to repetitive DNA. (A) Hmga enrichment at repeat families as defined by RepeatMasker. Log2 enrichments over the respective DBD-mutants (based on aggregated counts over all instances of a particular family) are shown for all families of a minimal length of 300nts and at least 10 occurrences in the genome. Average AT content is shown in green. In general, binding is increased with increasing AT content. The two seeming outliers, simple and satellite repeats, are heterogeneous in terms of AT content and also show a trend towards increased binding as a function of AT content when further subdivided into their corresponding different subtypes (S8B and S8C Fig).
(B) as in (A) but for all types of simple repeats of minimal length of 300nts and at least 10 occurrences in the genome.
(C) as in (A) but for all types of satellite repeats of minimal length of 300nts and at least 10 occurrences in the genome. GSAT_MM repeats represent major satellites (indicated by an arrow).
(TIFF)

S9 Fig Hmga1 mutant allele and add-back. (A) Allelic summary of Sanger sequencing of PCR products of the region targeted by CRISPR-Cas9 against the 3^rd^ exon of Hmga1. The downstream intron, which contains a premature stop-codon, is shown in yellow.
(B) WB of Hmga1 KO, Hmga1 add-back and parental ES cell lines (30 μgr of total cell protein extracts are loaded per lane), blotted against Hmga1.
(C) WB of Hmga1 KO and Hmga2 add-back ES cell lines (30 μgr of total cell protein extracts are loaded per lane), blotted against Hmga2.
(D) DBD-mutant-normalized log2 enrichments for Hmga1-2 either in the WT or in the Hmga1 KO background (DBD-mutant in the WT background is used for normalization in both cases). Each datapoint is calculated over a 10kb tiling window on chromosome 14. For better readability, top and bottom 1% of data range are not shown.
(E) Pearson correlation values for DBD-mutant-normalized Hmga1 and Hmga2 replicates and AT content in the Hmga1 KO background. All samples show a similar degree of positive correlation, in line with what we observe in the WT background.
(F) Scatterplots and Pearson correlation for one Hmga1 and one Hmga2 replicate and AT content in the Hmga1 KO background. Y-axis values show DBD-mutant (in the WT background)-normalized log2 enrichments.
(G) log2 changes in enrichment (over input) between WT and KO background for two replicates of Hmga1 (left) and Hmga2 (right) in 1kb tiling windows. There are no reproducible changes in binding between add-backs and WT.
(TIFF)

S10 Fig Clonogenicity and cell cycle analysis. (A) Clonogenicity assay of Hmga1 KO and parental cell line. Triplicate biological replicate staining with alkaline phosphatase for puripotency, 4 days after plating. No significant change in colony morphology or staining intensity can be seen.
(B) Cell cycle distribution profile of a representative WT (top) and Hmga1 KO sample (bottom), respectively.
(C) Percentage of parent gate and geometric mean of the Cell-Cycle Violet dye (d = day).
(D) Average GC-corrected read ratio per chromosome of WT and KO (see Materials and methods for details), indicating that the karyotype of both cell lines is identical.
(TIFF)

S11 Fig Transcriptomic analysis in the Hmga1 KO background at RefSeq genes. (A) Transcriptomic comparison of Hmga1 and Hmga2 add-backs vs. parental WT cell line at the gene level. Gene names are indicated if the gene is significantly differentially expressed (adjusted p-value < 0.01 and absolute fold-change of at least 2).
(B) Correlation heatmap of RNA-seq samples illustrating the very high correlation between all samples (colour indicates Pearson correlation).
(C) Scatterplots showing reproducibility of the transcriptional differences between 2 different KO, Hmga1 addback or Hmga2 addback samples and their corresponding WT samples (sequenced on the same lane). The significantly changing genes are shown in red.
(D) Scatterplots and Pearson correlations for all RNA-seq samples, illustrating the high similarity between all samples.
(TIFF)

S12 Fig Sashimi plot of the Hmga2 gene. Sashimi plot (generated as described in Materials and methods) over the Hmga2 gene in the Hmga1 KO condition. Hmga2 gene expression is unchanged in the KO compared to WT.
(TIFF)

S13 Fig Transcript and protein detection in Hmga1 mutants. (A) The mutated Hmga1 locus described in S9A Fig gives rise to an aberrant transcript (loss of the splice donor site of exon 3, which is the second coding exon) which undergoes nonsense-mediated decay (NMD) due to a premature STOP codon and inclusion of a large portion of the third intron as a 3’ UTR. NMD is triggered by the presence of a UTR upstream of a spliceable intron [107]. If NMD was not occurring a longer mRNA should be detectable by PCR, which is not the case (see S13B Fig). Nevertheless, Sashimi plot analysis highlights that transcription of an alternative Hmga1 isoform is occurring: this transcript is however completely devoid of exon 3. This exon encodes (in frame with the upstream and downstream exons) the second AT-hook domain, which harbors the conserved nuclear localization signal (NLS) [108] and displays by far the strongest DNA binding of all DBDs [109,110]. For quantification of protein abundance related to this transcript see S13C Fig. The reference transcript (ENSMUST00000117600.7), which the exon numbering refers to, is marked by a red arrow.
(B) In accordance to the splice junctions highlighted in the Sashimi plot, a shorter mRNA transcript, and not a longer one, can be detected by PCR in the KO condition. The PCR primers used anneal to the first and the last coding exon over the following regions: GAGAATGAGCGAGTCGGGCTC and GATCACTGCTCCTCCTCAGAGG. Marker 1 Kb Plus DNA Ladder (Invitrogen).
(C) Western Blot with TBP (loading control) and Hmga1 recognizing antibodies against the indicated samples. By Western Blot, a shorter protein (expected ~ 8.8 kDa) that could originate from the shorter transcript described in S13A and S13B Fig can neither be detected in the cytosolic + free nuclear fraction (n.e.b. A = nuclear extraction buffer A, see Materials and methods for buffer composition) nor in the chromatin-bound fraction (n.e.b. B = nuclear extraction buffer B). A marker containing a 3.5 kDa band was used to make sure that such a protein would be detected. We conclude that any potential protein originating from a transcript lacking exon 3 is unstable due to mis-localization (secondary to the absence of the NLS). In contrast, the WT protein is present in the chromatin-bound fraction of both the parental and Hmga1 add-back (higher molecular weight is due to the presence of the biotin-tag) samples.
(TIFF)

S14 Fig Transcriptomic analysis in the Hmga1 KO background at repetitive regions in the genome. (A) Transcriptomic comparison of Hmga1 and Hmga2 add-backs vs. parental WT cell line at repetitive regions of the genome as defined by RepeatMasker, excluding repeats lying on the same or opposite strand of annotated transcripts (Materials and methods). Repeats were not quantified at the level of single repeat instances but on the level of Repeat masker repeat “names”. No significant changes can be detected (adjusted p-value < 0.01 and absolute fold-change of at least 2).
(B) Correlation heatmap of RNA-seq samples illustrating high similarity between samples on the level of repeats (colour indicates Pearson correlation). As in the case of genes (S11B Fig), the most noticeable differences are between batches (a, b and c) rather than between samples.
(C) Scatterplots showing reproducibility for the indicated samples of the transcriptional differences at repeats between 2 different replicates and the corresponding WT samples. In agreement with the absence of significant changes in (A) and Fig 5E, there are no reproducible changes between replicates. Log2 count distributions were quantile-normalized before determining log2 differences due to differences in the shapes of the log2 count distributions between different samples.
(D) Scatterplots and Pearson correlations at repeats for all samples.
(TIFF)

S15 Fig Phenotype of differentiated cells. (A) WT (left) and Hmga1 KO (right) embryoid bodies 2 days after retinoic acid addition. No apparent differences can be detected.
(B) WT (left) and Hmga1 KO (right) neuronal progenitors at day 6 after plating, which marks the start of synaptic firing [52]. No apparent differences can be detected.
(C) WT (left) and Hmga1 KO (right) terminal neurons at day 10 after plating, when pruning has occurred and neurons are mature and stable [52]. No apparent differences can be detected.
(D) DAPI staining of nuclei of WT (left) and Hmga1 KO (right) plated neuronal progenitors. No apparent differences can be detected.
(TIFF)

S16 Fig Strategy and phenotype of genic deletion of Hmga1. (A) Strategy used for obtaining alleles carrying a “full-deletion” of the coding portion of Hmga1. Indicated are the location of designed sgRNAs and primers used for PCR screening of the single cell clones. Thicker boxes stand for the coding portion of Hmga1 exons.
(B) Sequencing results from the PCR product of the “external PCR” (see S16A Fig). Top: 3’ UTR junction. Bottom: 5’ UTR junction.
(C-E) PCRs showing presence/absence of respectively, from left to right, the external and internal Hmga1 regions (see S16A Fig) and an unrelated control region (see Materials and methods) for the indicated samples. In the WT background the external PCR (C) is too big to be amplified with the cycling conditions set. Sample loading in (E) is the same as in (D). A 100bp ladder (ThermoFisher, 15628019) was used, with reference bands at 2000, 1500, and 600 bp.
(F) WB on the indicated cell lines. Loading control and Hmga1-specific antibody staining highlight absence of Hmga1 in the KO and “full-deletion” clones.
(G) Sashimi plot over the Hmga1 locus for WT and Hmga1 “full-deletion” replicates, showing absence of transcription from the deleted locus.
(TIF)

S17 Fig Transcriptomic analysis in the Hmga1 full-deletion background. (A) Transcriptomic comparison of Hmga1 full-deletion vs. parental cell line at the gene level. Gene names are indicated if the gene is significantly differentially expressed (adjusted p-value < 0.01 and absolute fold-change of at least 2).
(B) Transcriptomic comparison of Hmga1 full-deletion vs. parental cell line at repetitive regions of the genome as defined by RepeatMasker, excluding repeats lying on the same or opposite strand of annotated transcripts (Materials and methods). Repeat elements show no significant changes (adjusted p-value < 0.01 and absolute fold-change of at least 2). Quantification was performed on the level of RepeatMasker repeat “names”.
(C)-(D) Scatterplots and Pearson correlations for all RNA-seq samples, illustrating the very high similarity between all samples at genes (C) and the high similarity at repeats (D).
(E) Scatterplots showing reproducibility of the transcriptional differences in the pairwise comparisons of WT and KO samples (all sequenced on the same lane). The significantly changing genes are shown in red.
(TIFF)

S18 Fig Sashimi plot of the Uty gene. Sashimi plot over the only gene significantly changed in both Hmga1 KO and full-deletion clones. Depicted are reads in the WT and full-deletion condition over the *Uty* gene, which already in the WT condition is transcribed at very low levels (note the missing exon-exon junctions). For comparison with the expression levels of a transcript that is efficiently translated see S16G.
(TIFF)
