# Supplementary material for: Binding of high mobility group A proteins to the mammalian genome occurs as a function of AT-content
Source: PLoS Genet. 2017 Dec 21;13(12):e1007102. doi: 10.1371/journal.pgen.1007102 (PMC5756049; doi:10.1371/journal.pgen.1007102)
Supplement: S1 Text — (DOCX) [file pgen.1007102.s001.docx]

**HMGA1**

MSESGSKSSQPLASKQEKDGTEKRGRGRPRKQPPVSPGTALVGSQKEPSEVPTPKRPRGRPKGSKNKGAAKTRKVTTAPGRKPRGRPKKLEKEEEEGISQESSEEEQ

**HMGA2**

MSARGEGAGQPSTSAQGQPAAPVPQKRGRGRPRKQQQEPTCEPSPKRPRGRPKGSKNKSPSKAAQKKAETIGEKRPRGRPRKWPQQVVQKKPAQETEETSSQESAEED

**GFP**

MVSKGEELFTGVVPILVELDGDVNGHKFSVSGEGEGDATYGKLTLKFICTTGKLPVPWPTLVTTLTYGVQCFSRYPDHMKQHDFFKSAMPEGYVQERTIFFKDDGNYKTRAEVKFEGDTLVNRIELKGIDFKEDGNILGHKLEYNYNSHNVYIMADKQKNGIKVNFKIRHNIEDGSVQLADHYQQNTPIGDGPVLLPDNHYLSTQSALSKDPNEKRDHMVLLEFVTAAGITLGMDELYK

**MutHMGA1**

MSESGSKSSQPLASKQEKDGTEKRGCGCPRKQPPVSPGTALVGSQKEPSEVPTPKRPCGRPKGSKNKGAAKTRKVTTAPGRKPCGCPKKLEKEEEEGISQESSEEEQ

**MutHMGA2**

MSARGEGAGQPSTSAQGQPAAPVPQKRGCGCPRKQQQEPTCEPSPKRPCGCPKGSKNKSPSKAAQKKAETIGEKRPCGCPRKWPQQVVQKKPAQETEETSSQESAEED
