# Supplementary figures and images for: Binding of high mobility group A proteins to the mammalian genome occurs as a function of AT-content
Source: PLoS Genet. 2017 Dec 21;13(12):e1007102. doi: 10.1371/journal.pgen.1007102 (PMC5756049; doi:10.1371/journal.pgen.1007102)

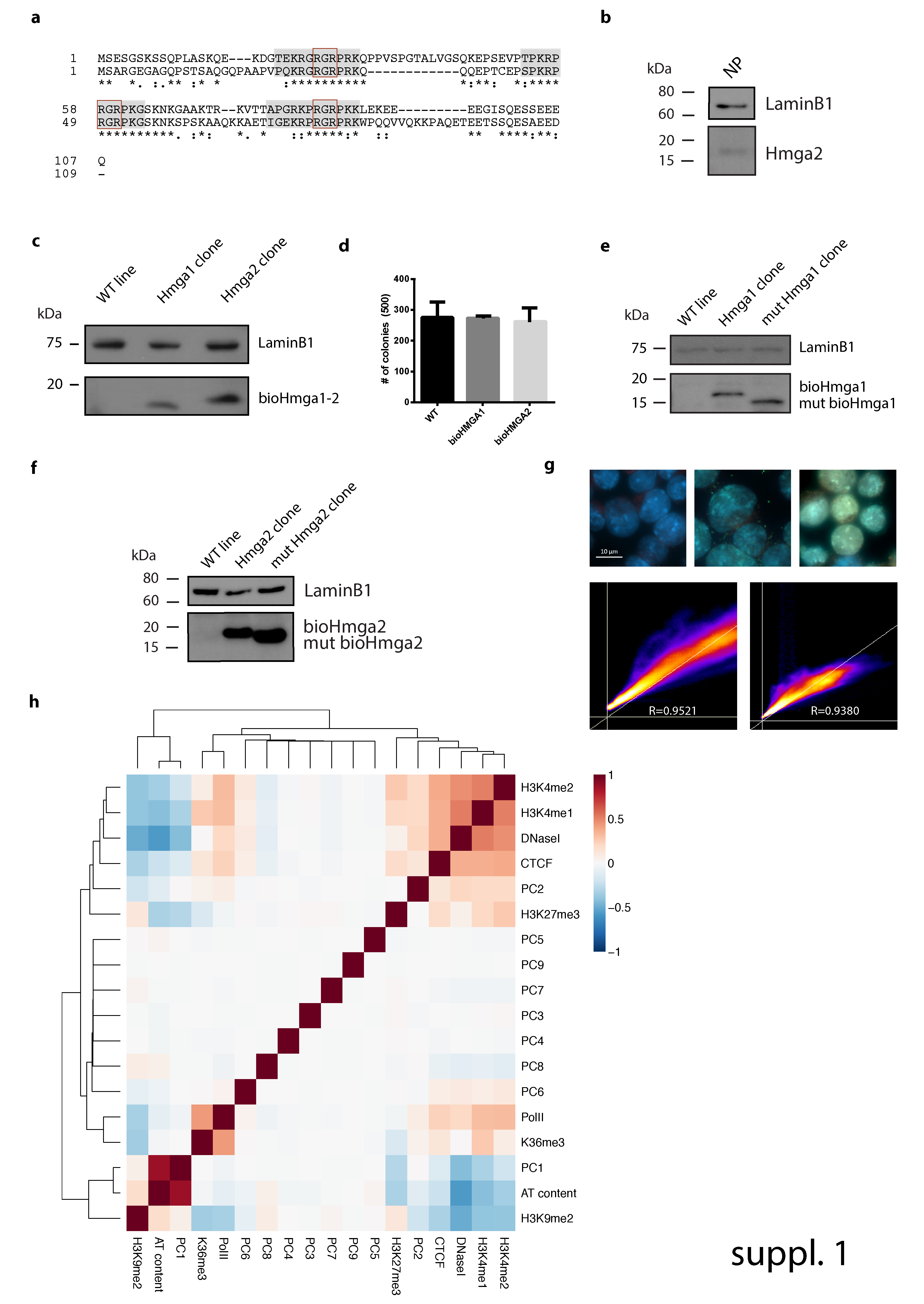

Supplement: S1 Fig — (A) Protein alignment between Hmga1 (top row) and Hmga2 (bottom row) obtained with Uniprot through Clustal Omega [106]. Highlighted in grey are the DNA-binding domains, which show notable levels of conservation as assessed by the number of identical (*), highly similar (:) and similar (.) amino acids. The conserved regions where bulky and positively charged Arg residues were mutated to Cys are shown in red. (B) WB with anti-Hmga2 Ab of whole cell lysate from cells differentiated to the neuronal progenitor stage. A band is visible at the expected height for Hmga2 (for comparison with bioHmga2 see S1F Fig). (C) WB with anti-SAV conjugated HRP of whole-cell lysate from parental cell line and cells expressing Hmga1 and Hmga2. Although slightly different, expression levels are comparable between both proteins. (D) Clonogenicity assay for the indicated cell lines showing similar pluripotent potential for cell lines expressing the biotinylated constructs. Displayed are the mean and standard deviation of 3 replicate counts of alkaline phosphatase positive colonies 5 days after plating to clonal density (number of single cells in brackets). None of the engineered lines show a significant difference to the parental cell line (one-way ANOVA, Bonferroni’s multiple comparison correction, CI 95%). (E) WB with anti-SAV conjugated HRP of whole cell lysate from parental cell line and cells expressing Hmga1 or mutated Hmga1. In the last lane, a lower molecular-weight band (due to a smaller mass of the side chains of the cysteine residues as compared to the WT arginines) representing mutated bioHmga1 is visible and shows a comparable expression level to bioHmga1. Blotting with SAV was chosen because the mutated bioHmga1 protein runs at the same position as the untagged Hmga1. (F) WB with anti Hmga2 Ab of whole-cell lysate from parental cell line and cells expressing Hmga2 or mutated Hmga2. As expected from the lack of mRNA signal (Fig 1B), Hmga2 is not expressed in ESC. A lower molecu [file pgen.1007102.s002.tif]

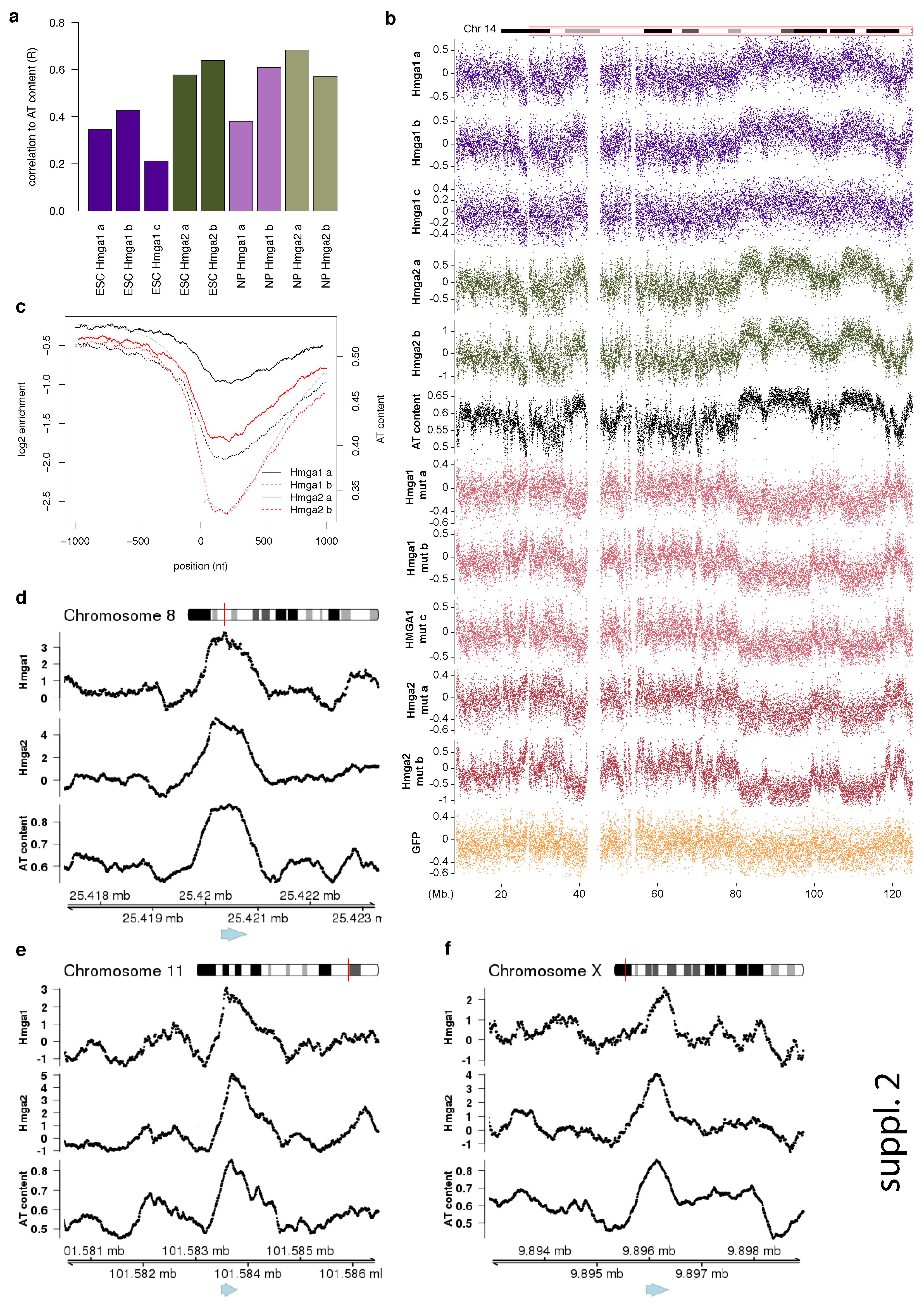

Supplement: S2 Fig — (A) Barplot showing genome-wide correlations for all Hmga1 and Hmga2 replicates in ESC and NPs (log2 enrichments over input) with respect to genomic AT content. (B) Log2 enrichments over input for the depicted samples on chromosome 14. Each dot represents the log2 enrichment of IP over input in a window of size 10kb. Gaps indicate regions with low mappabilty (below 80%). Top and bottom 1% of data range are not shown to enhance readability. Data for replicate c of Hmga1 and Hmga1 DBD-mutant was obtained using a different ChIP protocol and highlights the robustness of the results (see Materials and methods). (C) Hmga binding is depleted at CpG islands as it follows AT content. Average profiles were calculated relative to CpG island starts (nt). Enrichment denotes enrichment over the corresponding DBD-mutant. AT content is shown in grey (dashed line). AT content is not shown directly around CpG island starts (position 0) since the start position is by definition a CpG, resulting in an artificial local dip in AT content. All values denote a running mean over 51 nucleotides. (D)-(E)-(F) Single locus tracks of log2 enrichments over the respective DBD-mutants at three (TA)n simple repeats with strong binding. To increase coverage, the counts of all Hmga1 samples, all Hmga2 samples, all Hmga1 DBD-mutant samples and all Hmga2 DBD-mutant samples were separately pooled and aggregated in a running window of 401 nts before determining enrichments (see Materials and methods). The locations of the (TA)n repeats are marked by arrows. (TIF) [file pgen.1007102.s003.tif]

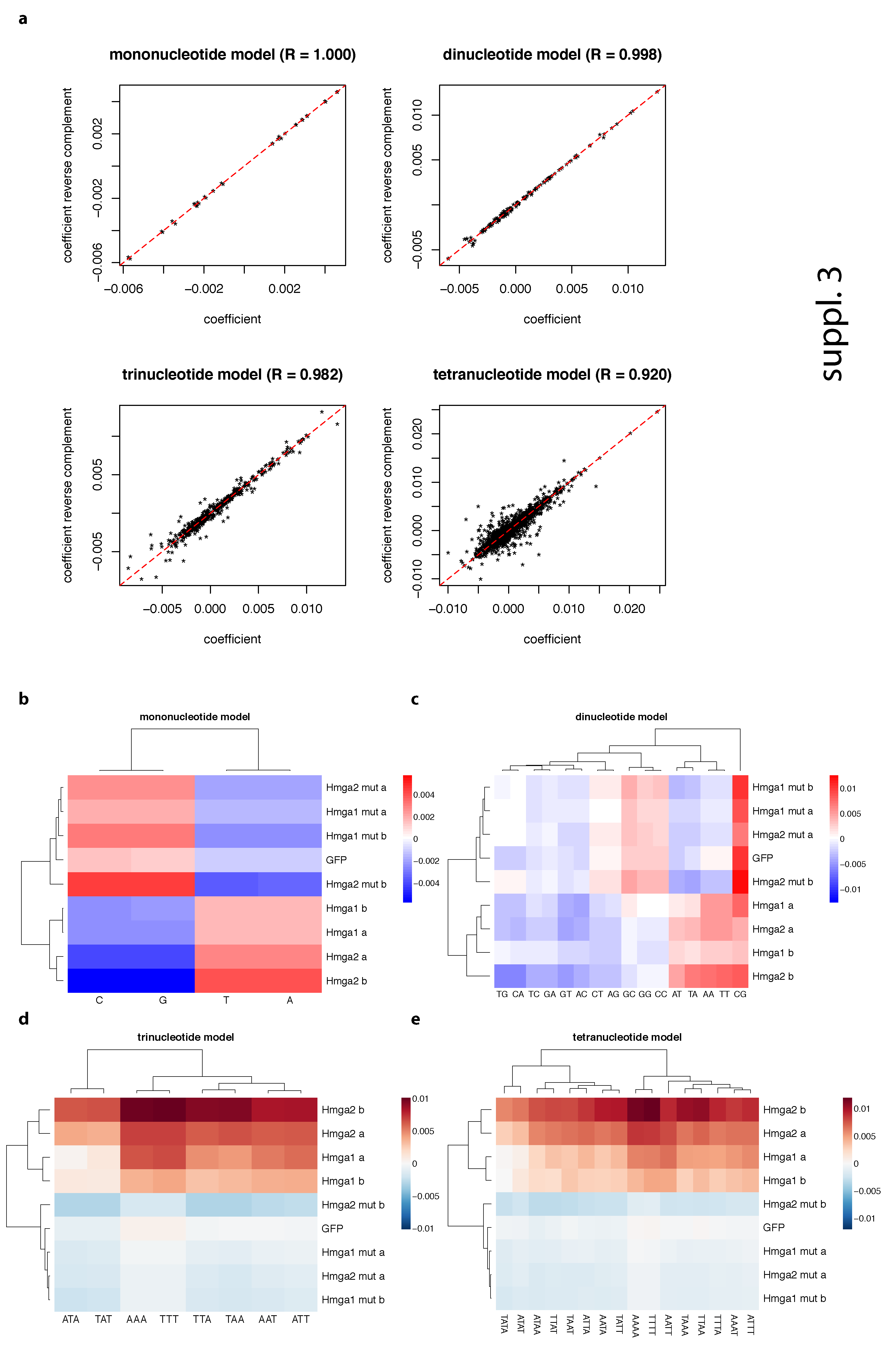

Supplement: S3 Fig — (A) Inferred ridge regression coefficients are internally consistent. Shown are scatter plots of the inferred coefficients for each mono-, di-, tri- or tetranucleotide versus the coefficients of its reverse complements for the respective models. As the ChIP-seq data does not contain any strand information, the inferred coefficient for a nucleotide should be very similar to the coefficient of its reverse complement (the nucleotide frequencies that act as predictors for the ridge regression are determined from the plus strand sequence only). This is indeed the case as evidenced by the high Pearson correlation coefficients (R). (B)-(C) Inferred ridge regression coefficients for the mono- (left) and dinucleotides (right) for each sample. For Hmga WT proteins high coefficients are apparent for A and T nucleotides and combinations thereof. The “CG” dinucleotide coefficients are high in all samples which may reflect the unspecific recruitment to accessible regions, which tend to be CpG- and GC-rich [47]. (D)-(E) Inferred ridge regression coefficients for tri- (left) and tetranucleotides (right) consisting of only As and/or Ts. The inferred contributions of the different tri- or tetranucleotides are highly comparable. Nonetheless, there is a subtle, but reproducible preference of polyA or polyT stretches over polyAT or polyTA stretches. However, this and other subtle differences are too small to substantially improve the predictive power of the tri- or tetranucleotide model over the simple mononucleotide model (Fig 2C). (TIF) [file pgen.1007102.s004.tif]

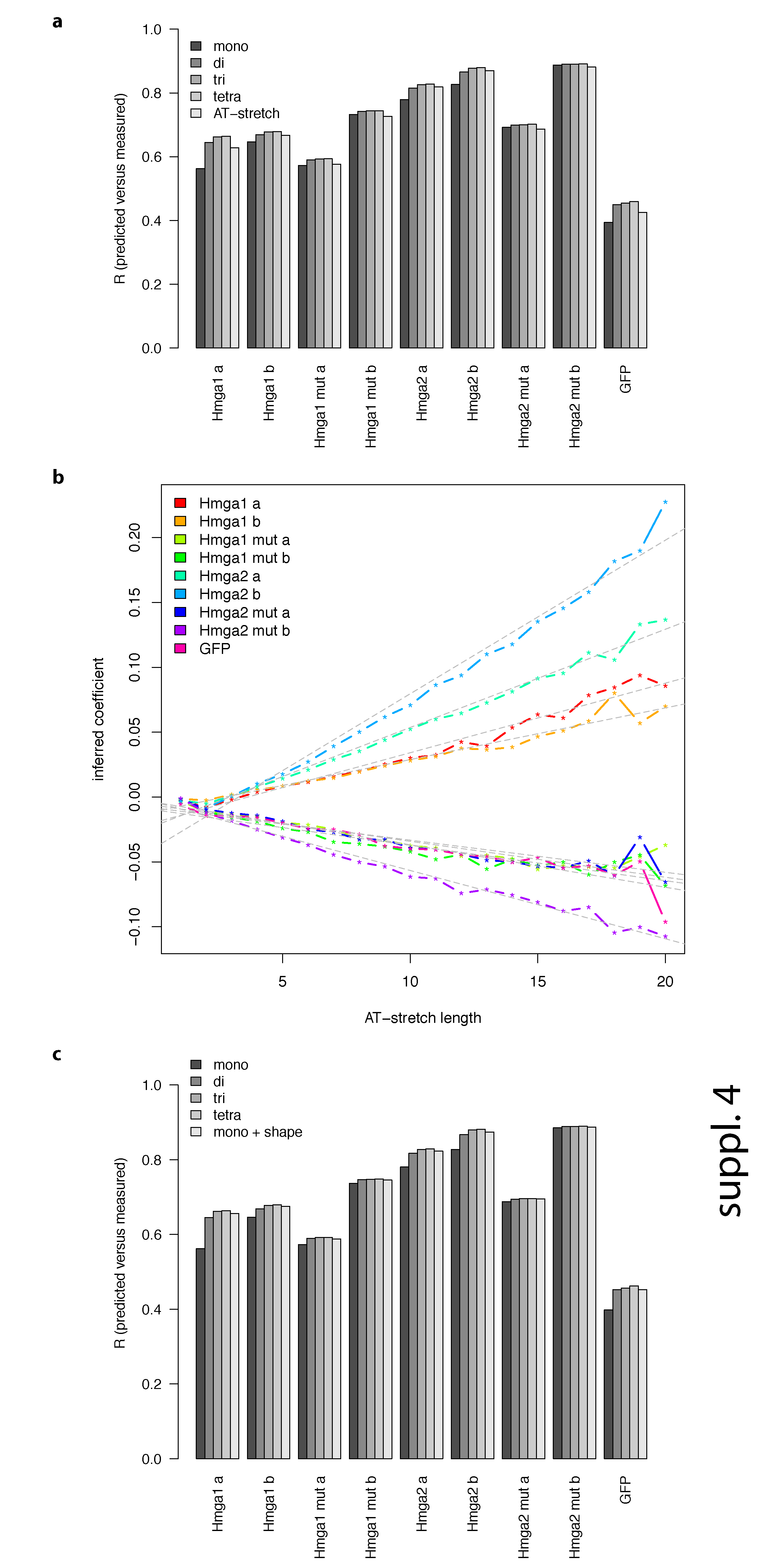

Supplement: S4 Fig — (A) The improvement in predictive power of the AT-stretch model over the simple mononucleotide model is small. R stands for the Pearson correlation coefficient. (B) Inferred coefficients of AT-stretches of length L scale linearly with L. This is in agreement with a model where the addition of one extra A or T leads to a constant increase in binding, which is in agreement with the predictions of the simple mononucleotide model. (C) The improvement in predictive power of a model that incorporates local structural features of the DNA (see Materials and methods) in addition to mononucleotide frequencies is also only minor. R stands for the Pearson correlation coefficient. (TIF) [file pgen.1007102.s005.tif]

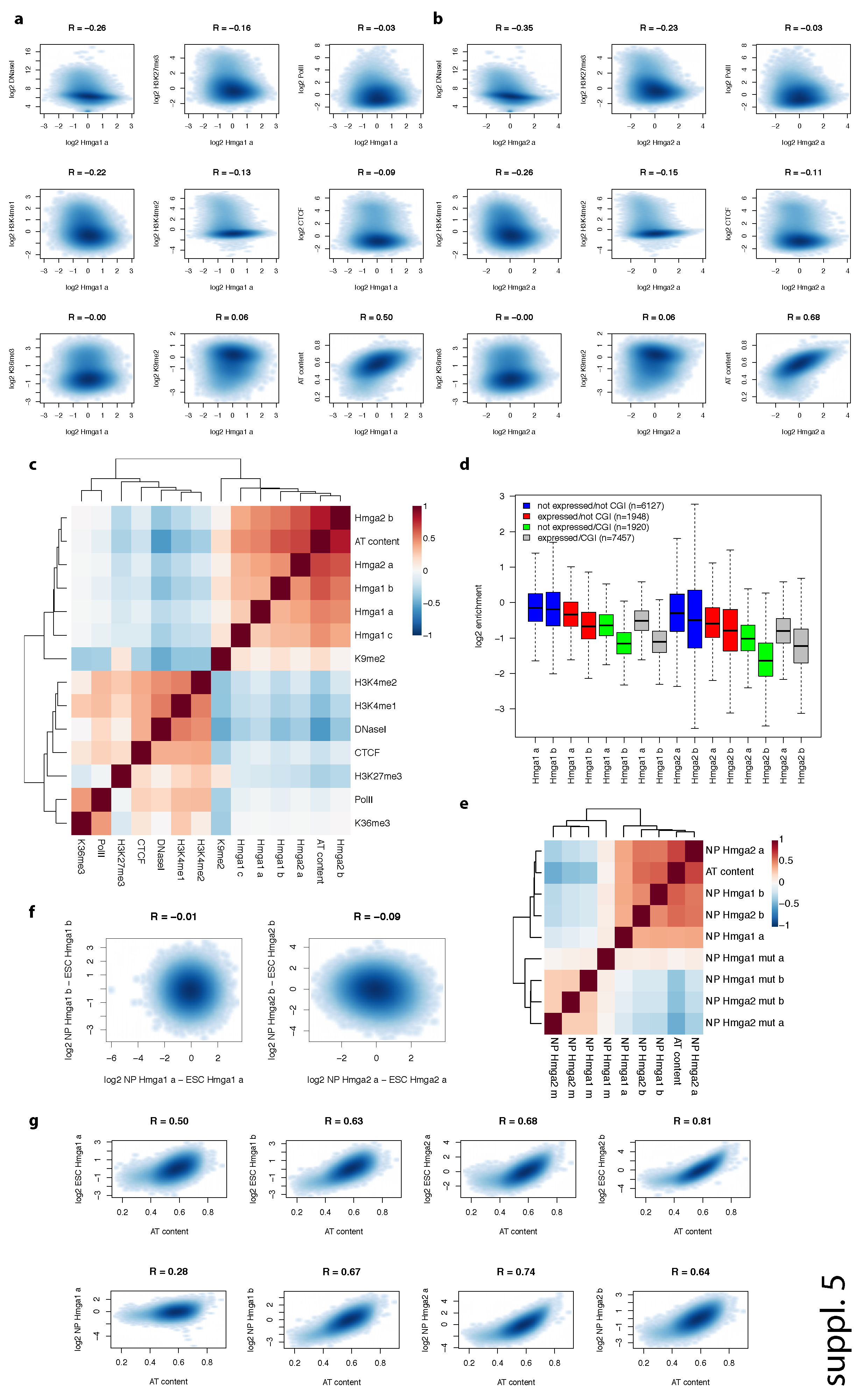

Supplement: S5 Fig — (A) Scatterplots of log2 enrichments over DBD-mutant versus chromatin features and AT content for a representative Hmga1 replicate (1kb tiling windows, R: Pearson correlation coefficient). (B) Same as in (A) for a representative Hmga2 replicate. (C) Correlation heatmap of cut frequency of DNaseI, AT content, chromatin marks, CTCF and bioChIP samples (log2 enrichments over DBD-mutant, 1kb tiling windows, colours indicate the Pearson correlation coefficient). Hmga1-2 correlate most strongly with AT content and form a separate cluster, in agreement with the PCA. (D) Distribution of DBD-mutant-normalized log2 enrichments for the indicated samples over promoters stratified into different categories: promoters overlapping (CGI) or not overlapping a CpG island (not CGI), expressed or not expressed. Hmga1 and Hmga2 enrichments are generally lower at CpG island promoters. Non-expressed non-CpG island promoters (blue boxplots) appear to have slightly larger enrichments than expressed non-CpG island promoters (in red). (E) Correlation heatmap of log2 enrichments over input for all NP samples, showing that as in ESC, WT and DBD-mutant samples form separate clusters and WT samples show good correlations to AT content. (F) Scatterplot and Pearson’s correlation of the log2 difference between NP and ESC enrichment values (over DBD-mutant) for two replicates of Hmga1 (left) and Hmga2 (right) over 1kb tiling windows indicating that there are no reproducible differences in binding between ESC and NPs. (G) Scatterplot and Pearson’s correlation of Hmga1 and Hmga2 samples versus AT content in ESC (top row) and NPs (bottom row), illustrating the nature of the positive correlation for all samples (1kb windows, log2 enrichments over DBD-mutant). (TIF) [file pgen.1007102.s006.tif]

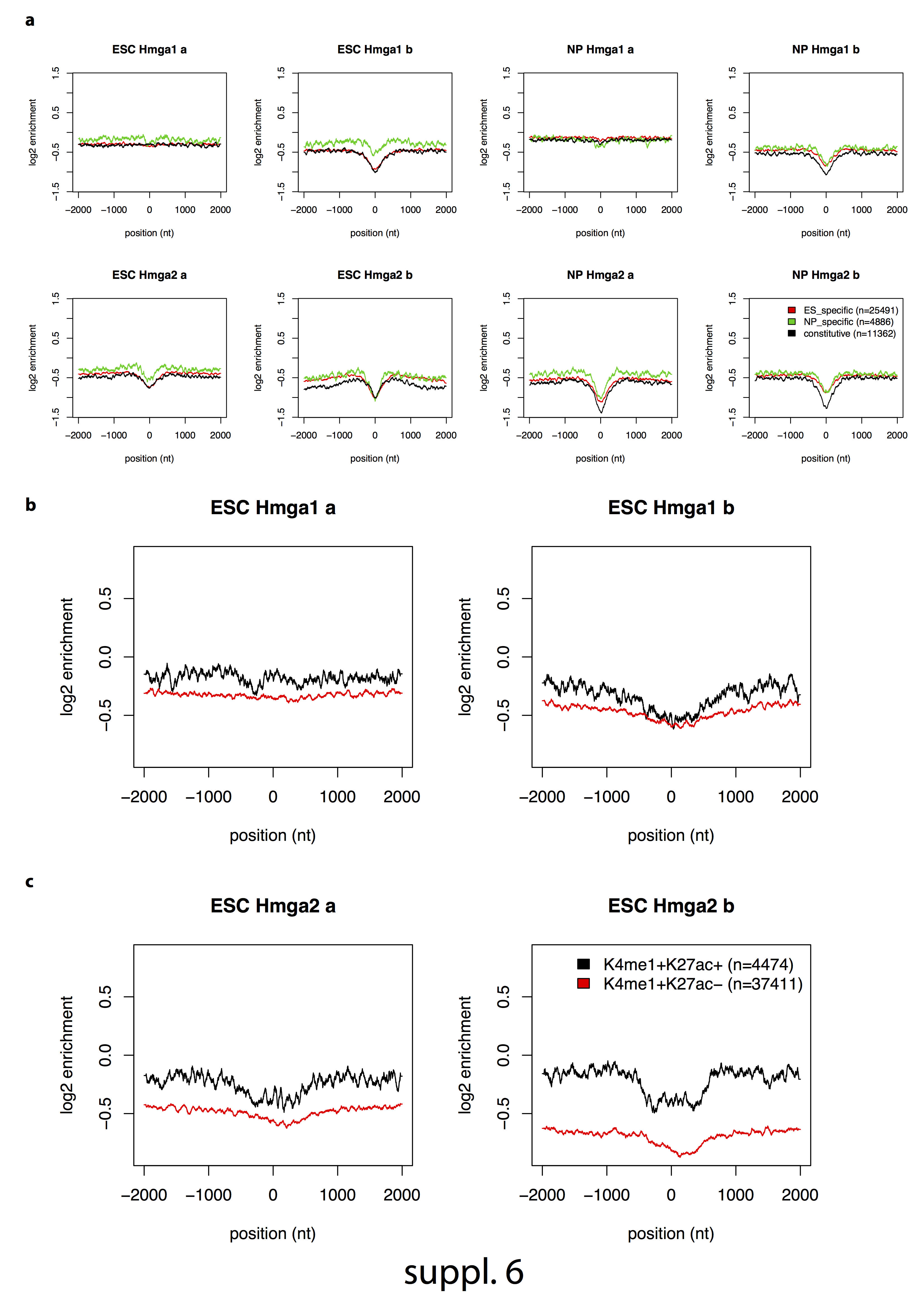

Supplement: S6 Fig — (A) Average profiles at LMRs [48] of log2 enrichments over DBD-mutant for two replicates of Hmga1 (top row) and Hmga2 (bottom row) in ESC and NP. Values were smoothed over 51 nts. (B) Average profiles at primed enhancers (marked by K4me1 and not marked by K27ac (K4me1+K27ac-)) and active (marked by both K4me1 and K27ac (K4me1+K27ac+)) enhancers for two Hmga1 replicates in ESC. Enrichments denote enrichment over the respective DBD-mutants. (C) Same as in (B) for two Hmga2 replicates in ESC. (TIFF) [file pgen.1007102.s007.tiff]

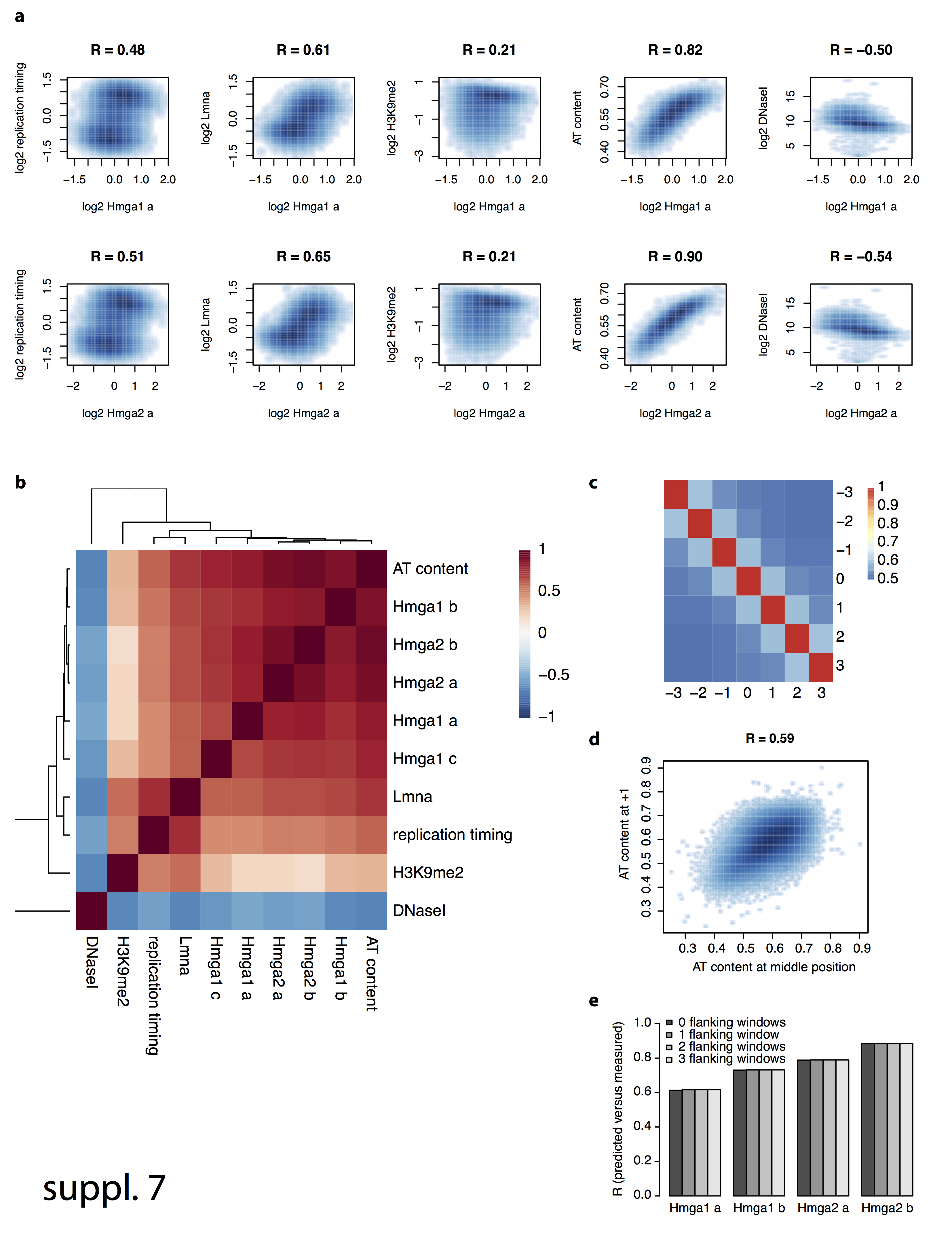

Supplement: S7 Fig — (A) Scatterplot of log2 enrichment values over DBD-mutant in 10kb tiling windows for one representative Hmga1 (top) and Hmga2 (bottom) replicate versus the indicated heterochromatic features, AT content or DNaseI cut frequency (same data as in Fig 4C). R denotes the Pearson correlation coefficient. (B) Genome-wide correlation heatmap of all measures in (A) and all ESC Hmga1 and Hmga2 replicates (10kb tiling windows, colours indicate the Pearson correlation coefficient). (C) Correlation heatmap of AT content in the set of neighbouring 1kb windows used for linear modelling (see Materials and methods). 0 refers to the central window, +/- n to the n-th neighbouring window downstream or upstream, respectively. The correlation between neighbouring windows is not very strong, thus containing potential additional information for linear modelling. (D) Example scatterplot of AT content in the central versus the directly neighbouring downstream window (+1). R indicates Pearson correlation coefficient. (E) Pearson correlation of predicted log2 Hmga enrichments (over DBD-mutant) versus measured values for linear models that use only the AT content of the window itself or the AT content of the window itself as well as of 1–3 neighbouring windows both up- and downstream. There is no substantial increase in predictive power when the AT content of neighbouring windows is taken into account. (TIFF) [file pgen.1007102.s008.tiff]

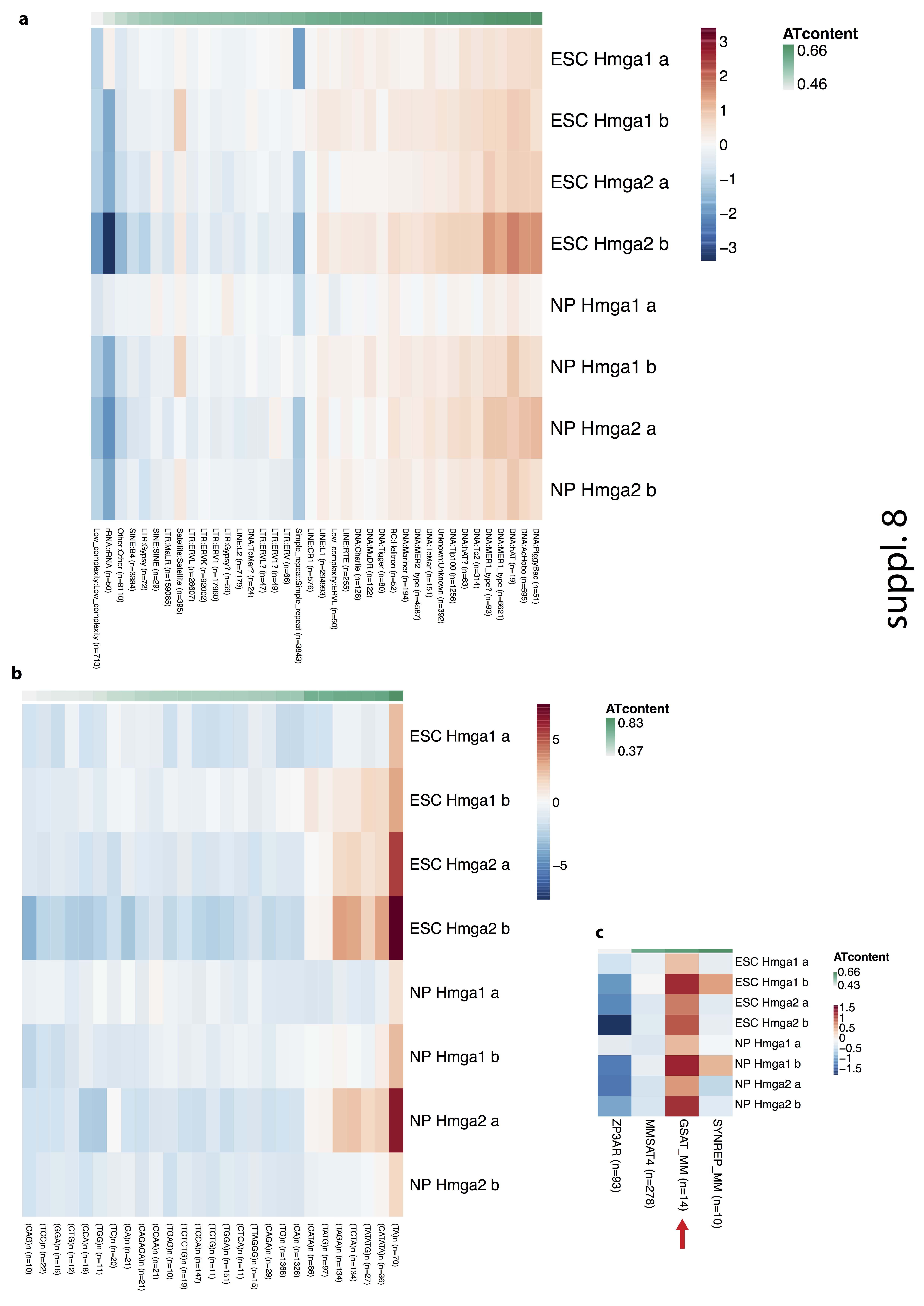

Supplement: S8 Fig — (A) Hmga enrichment at repeat families as defined by RepeatMasker. Log2 enrichments over the respective DBD-mutants (based on aggregated counts over all instances of a particular family) are shown for all families of a minimal length of 300nts and at least 10 occurrences in the genome. Average AT content is shown in green. In general, binding is increased with increasing AT content. The two seeming outliers, simple and satellite repeats, are heterogeneous in terms of AT content and also show a trend towards increased binding as a function of AT content when further subdivided into their corresponding different subtypes (S8B and S8C Fig). (B) as in (A) but for all types of simple repeats of minimal length of 300nts and at least 10 occurrences in the genome. (C) as in (A) but for all types of satellite repeats of minimal length of 300nts and at least 10 occurrences in the genome. GSAT_MM repeats represent major satellites (indicated by an arrow). (TIFF) [file pgen.1007102.s009.tiff]

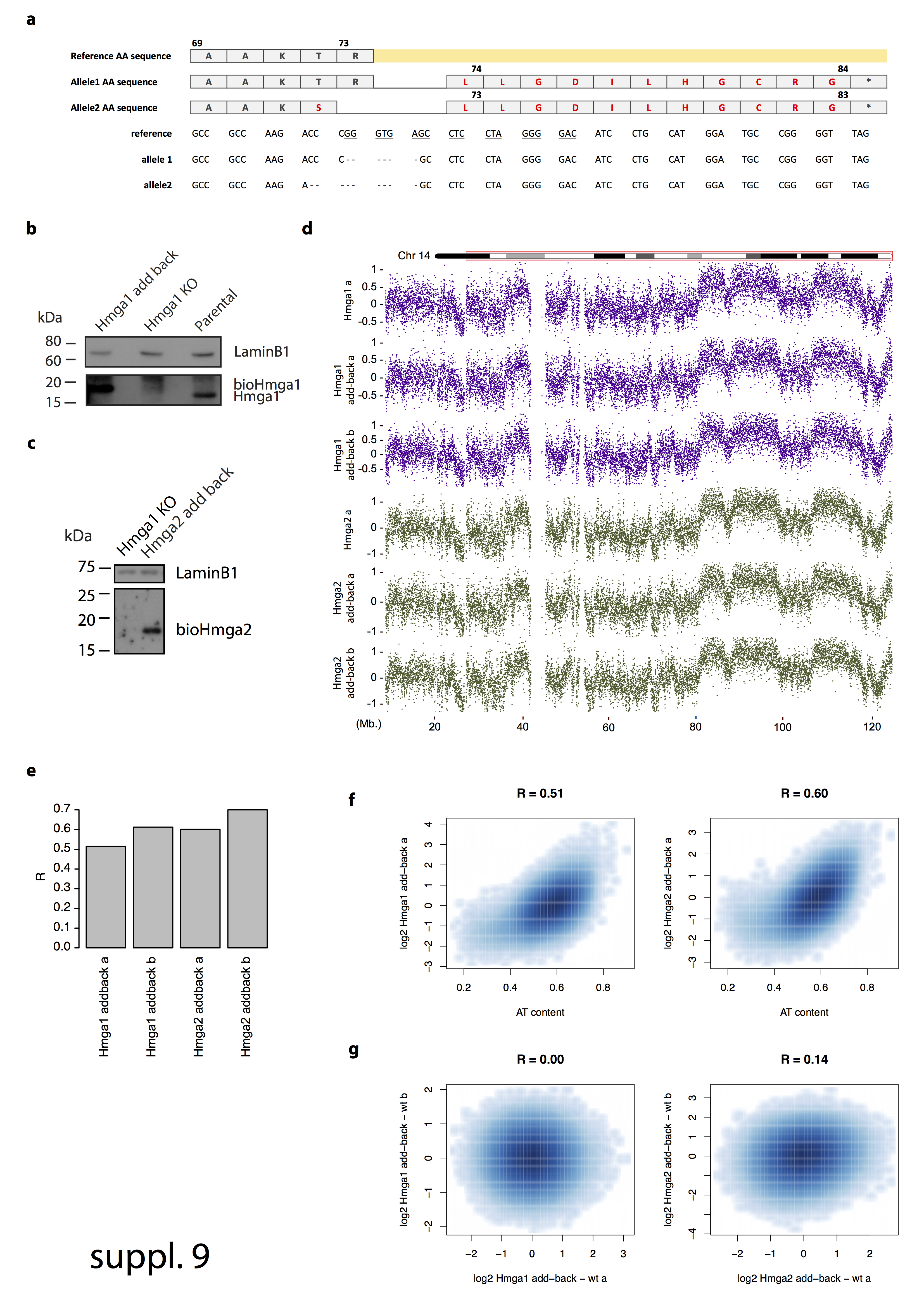

Supplement: S9 Fig — (A) Allelic summary of Sanger sequencing of PCR products of the region targeted by CRISPR-Cas9 against the 3rd exon of Hmga1. The downstream intron, which contains a premature stop-codon, is shown in yellow. (B) WB of Hmga1 KO, Hmga1 add-back and parental ES cell lines (30 μgr of total cell protein extracts are loaded per lane), blotted against Hmga1. (C) WB of Hmga1 KO and Hmga2 add-back ES cell lines (30 μgr of total cell protein extracts are loaded per lane), blotted against Hmga2. (D) DBD-mutant-normalized log2 enrichments for Hmga1-2 either in the WT or in the Hmga1 KO background (DBD-mutant in the WT background is used for normalization in both cases). Each datapoint is calculated over a 10kb tiling window on chromosome 14. For better readability, top and bottom 1% of data range are not shown. (E) Pearson correlation values for DBD-mutant-normalized Hmga1 and Hmga2 replicates and AT content in the Hmga1 KO background. All samples show a similar degree of positive correlation, in line with what we observe in the WT background. (F) Scatterplots and Pearson correlation for one Hmga1 and one Hmga2 replicate and AT content in the Hmga1 KO background. Y-axis values show DBD-mutant (in the WT background)-normalized log2 enrichments. (G) log2 changes in enrichment (over input) between WT and KO background for two replicates of Hmga1 (left) and Hmga2 (right) in 1kb tiling windows. There are no reproducible changes in binding between add-backs and WT. (TIFF) [file pgen.1007102.s010.tiff]

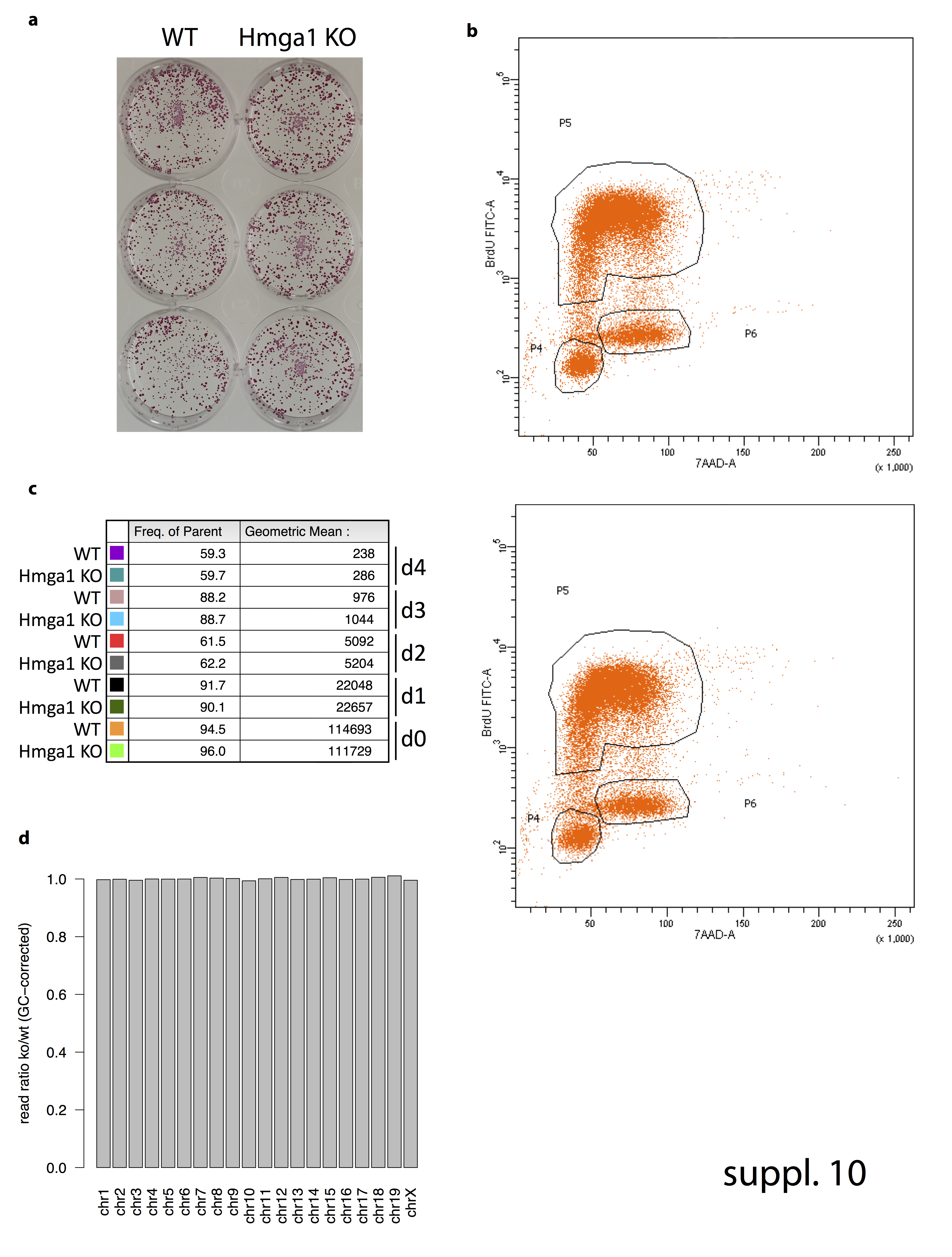

Supplement: S10 Fig — (A) Clonogenicity assay of Hmga1 KO and parental cell line. Triplicate biological replicate staining with alkaline phosphatase for puripotency, 4 days after plating. No significant change in colony morphology or staining intensity can be seen. (B) Cell cycle distribution profile of a representative WT (top) and Hmga1 KO sample (bottom), respectively. (C) Percentage of parent gate and geometric mean of the Cell-Cycle Violet dye (d = day). (D) Average GC-corrected read ratio per chromosome of WT and KO (see Materials and methods for details), indicating that the karyotype of both cell lines is identical. (TIFF) [file pgen.1007102.s011.tiff]

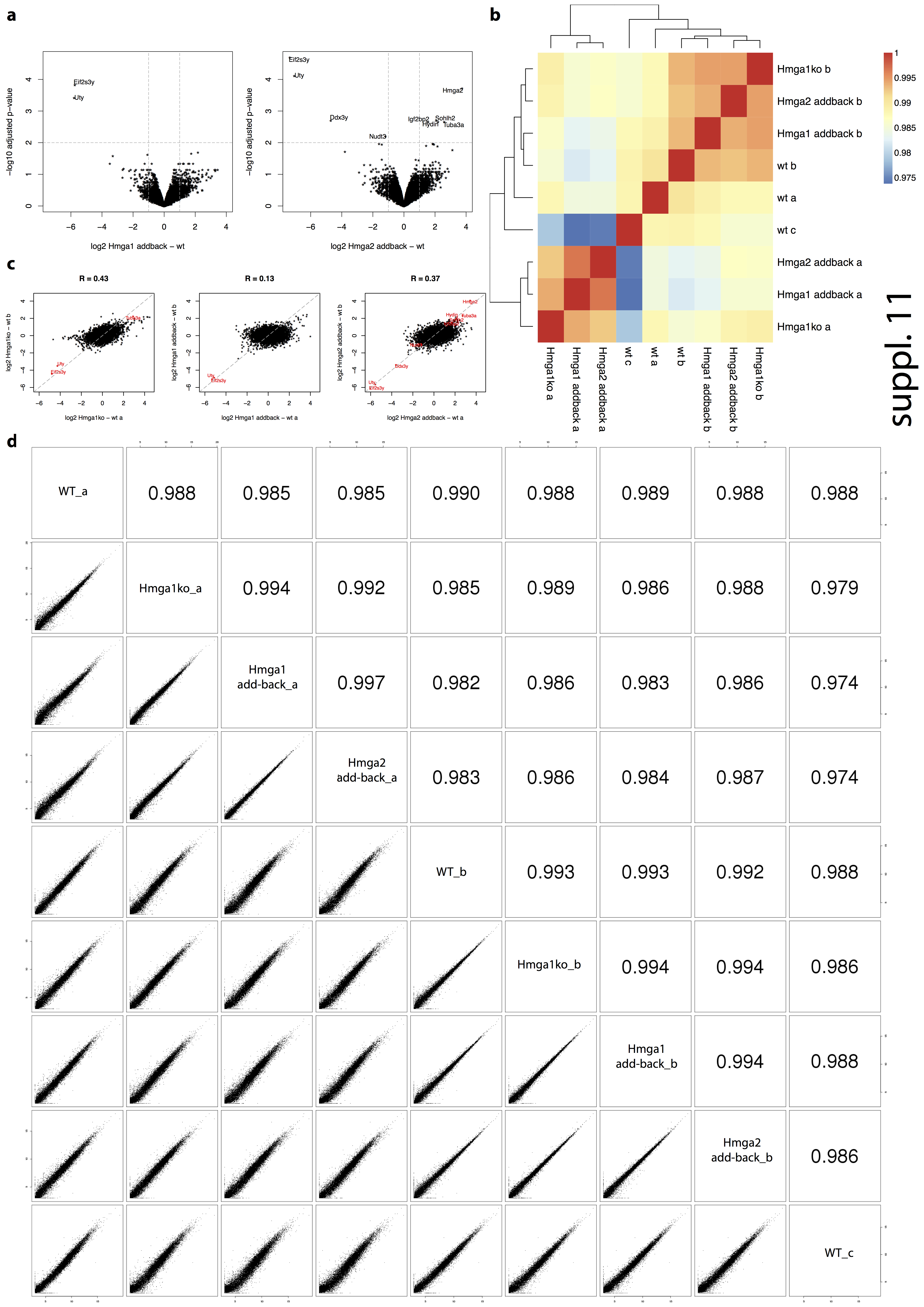

Supplement: S11 Fig — (A) Transcriptomic comparison of Hmga1 and Hmga2 add-backs vs. parental WT cell line at the gene level. Gene names are indicated if the gene is significantly differentially expressed (adjusted p-value < 0.01 and absolute fold-change of at least 2). (B) Correlation heatmap of RNA-seq samples illustrating the very high correlation between all samples (colour indicates Pearson correlation). (C) Scatterplots showing reproducibility of the transcriptional differences between 2 different KO, Hmga1 addback or Hmga2 addback samples and their corresponding WT samples (sequenced on the same lane). The significantly changing genes are shown in red. (D) Scatterplots and Pearson correlations for all RNA-seq samples, illustrating the high similarity between all samples. (TIFF) [file pgen.1007102.s012.tiff]

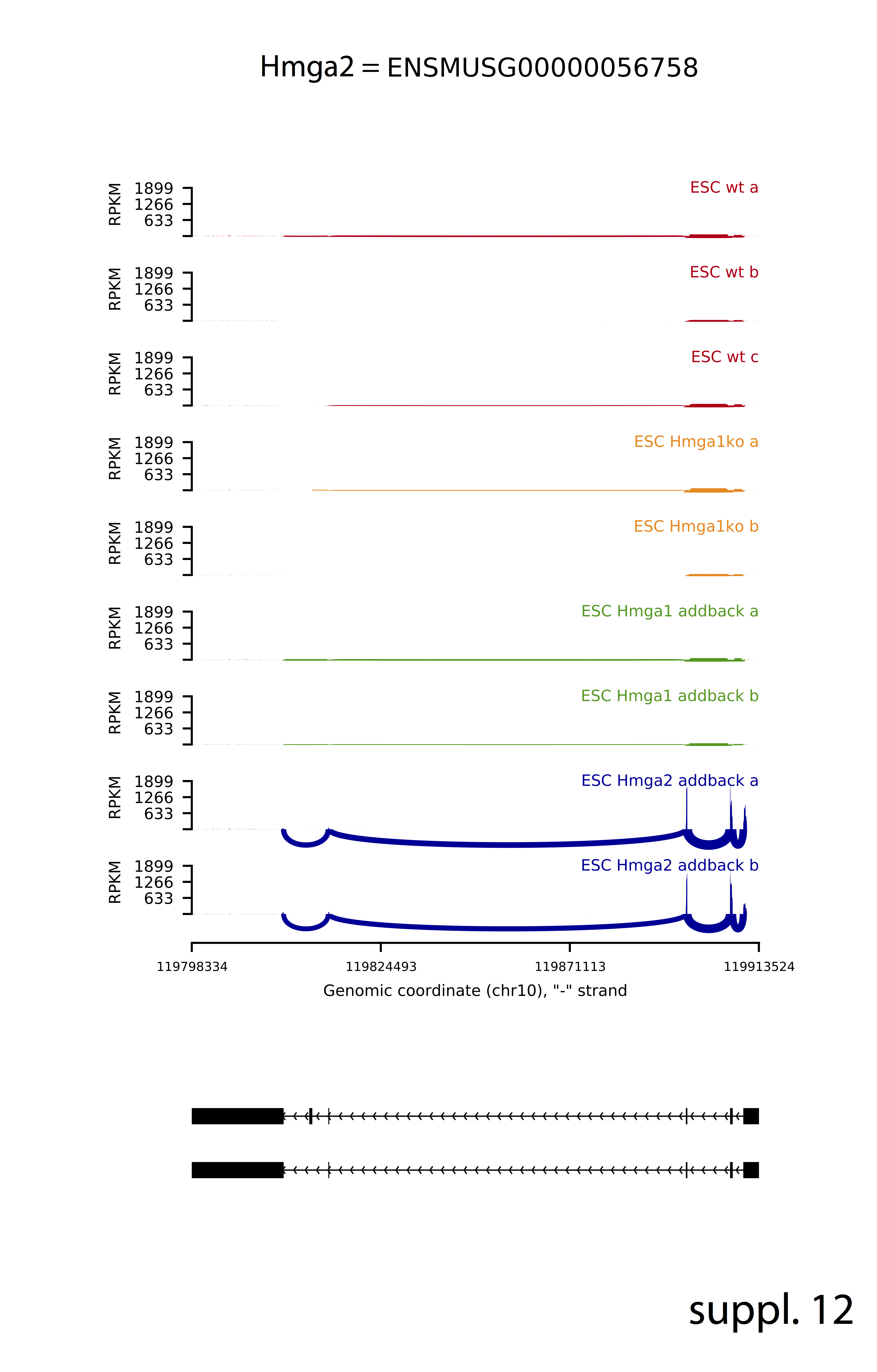

Supplement: S12 Fig — Sashimi plot (generated as described in Materials and methods) over the Hmga2 gene in the Hmga1 KO condition. Hmga2 gene expression is unchanged in the KO compared to WT. (TIFF) [file pgen.1007102.s013.tiff]

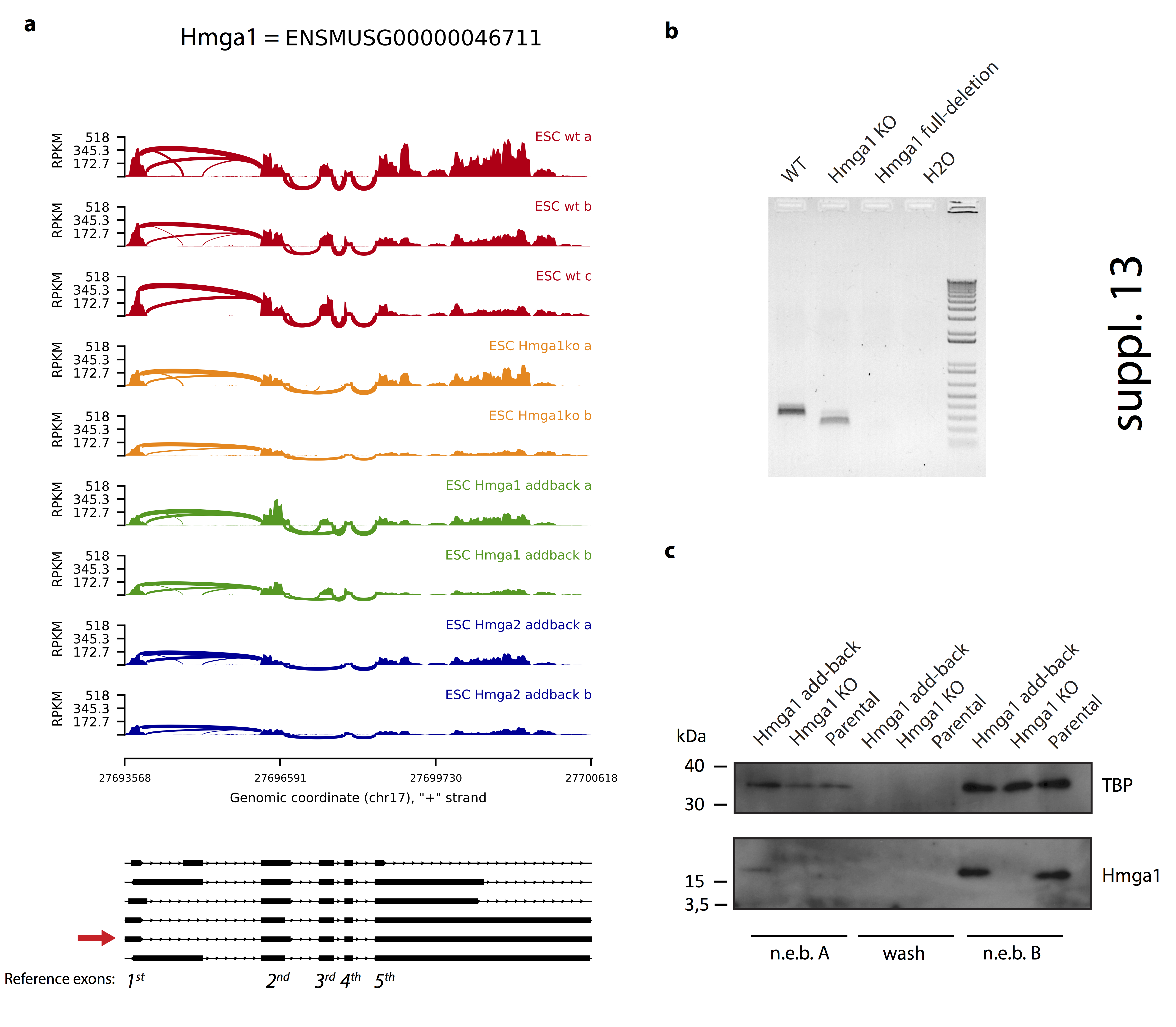

Supplement: S13 Fig — (A) The mutated Hmga1 locus described in S9A Fig gives rise to an aberrant transcript (loss of the splice donor site of exon 3, which is the second coding exon) which undergoes nonsense-mediated decay (NMD) due to a premature STOP codon and inclusion of a large portion of the third intron as a 3’ UTR. NMD is triggered by the presence of a UTR upstream of a spliceable intron [107]. If NMD was not occurring a longer mRNA should be detectable by PCR, which is not the case (see S13B Fig). Nevertheless, Sashimi plot analysis highlights that transcription of an alternative Hmga1 isoform is occurring: this transcript is however completely devoid of exon 3. This exon encodes (in frame with the upstream and downstream exons) the second AT-hook domain, which harbors the conserved nuclear localization signal (NLS) [108] and displays by far the strongest DNA binding of all DBDs [109,110]. For quantification of protein abundance related to this transcript see S13C Fig. The reference transcript (ENSMUST00000117600.7), which the exon numbering refers to, is marked by a red arrow. (B) In accordance to the splice junctions highlighted in the Sashimi plot, a shorter mRNA transcript, and not a longer one, can be detected by PCR in the KO condition. The PCR primers used anneal to the first and the last coding exon over the following regions: GAGAATGAGCGAGTCGGGCTC and GATCACTGCTCCTCCTCAGAGG. Marker 1 Kb Plus DNA Ladder (Invitrogen). (C) Western Blot with TBP (loading control) and Hmga1 recognizing antibodies against the indicated samples. By Western Blot, a shorter protein (expected ~ 8.8 kDa) that could originate from the shorter transcript described in S13A and S13B Fig can neither be detected in the cytosolic + free nuclear fraction (n.e.b. A = nuclear extraction buffer A, see Materials and methods for buffer composition) nor in the chromatin-bound fraction (n.e.b. B = nuclear extraction buffer B). A marker containing a 3.5 kDa band was used to make sure that such a protein would be [file pgen.1007102.s014.tiff]

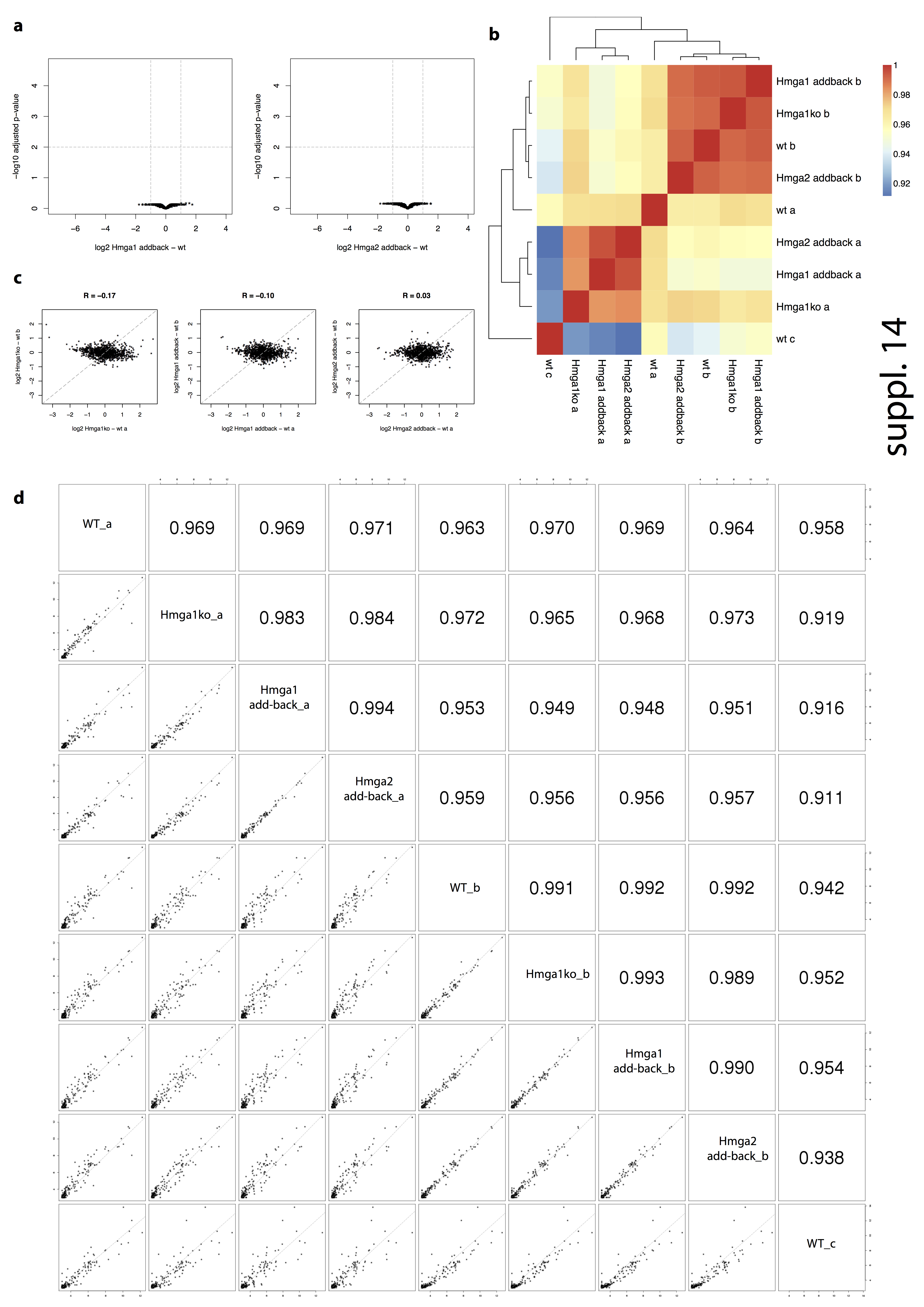

Supplement: S14 Fig — (A) Transcriptomic comparison of Hmga1 and Hmga2 add-backs vs. parental WT cell line at repetitive regions of the genome as defined by RepeatMasker, excluding repeats lying on the same or opposite strand of annotated transcripts (Materials and methods). Repeats were not quantified at the level of single repeat instances but on the level of Repeat masker repeat “names”. No significant changes can be detected (adjusted p-value < 0.01 and absolute fold-change of at least 2). (B) Correlation heatmap of RNA-seq samples illustrating high similarity between samples on the level of repeats (colour indicates Pearson correlation). As in the case of genes (S11B Fig), the most noticeable differences are between batches (a, b and c) rather than between samples. (C) Scatterplots showing reproducibility for the indicated samples of the transcriptional differences at repeats between 2 different replicates and the corresponding WT samples. In agreement with the absence of significant changes in (A) and Fig 5E, there are no reproducible changes between replicates. Log2 count distributions were quantile-normalized before determining log2 differences due to differences in the shapes of the log2 count distributions between different samples. (D) Scatterplots and Pearson correlations at repeats for all samples. (TIFF) [file pgen.1007102.s015.tiff]

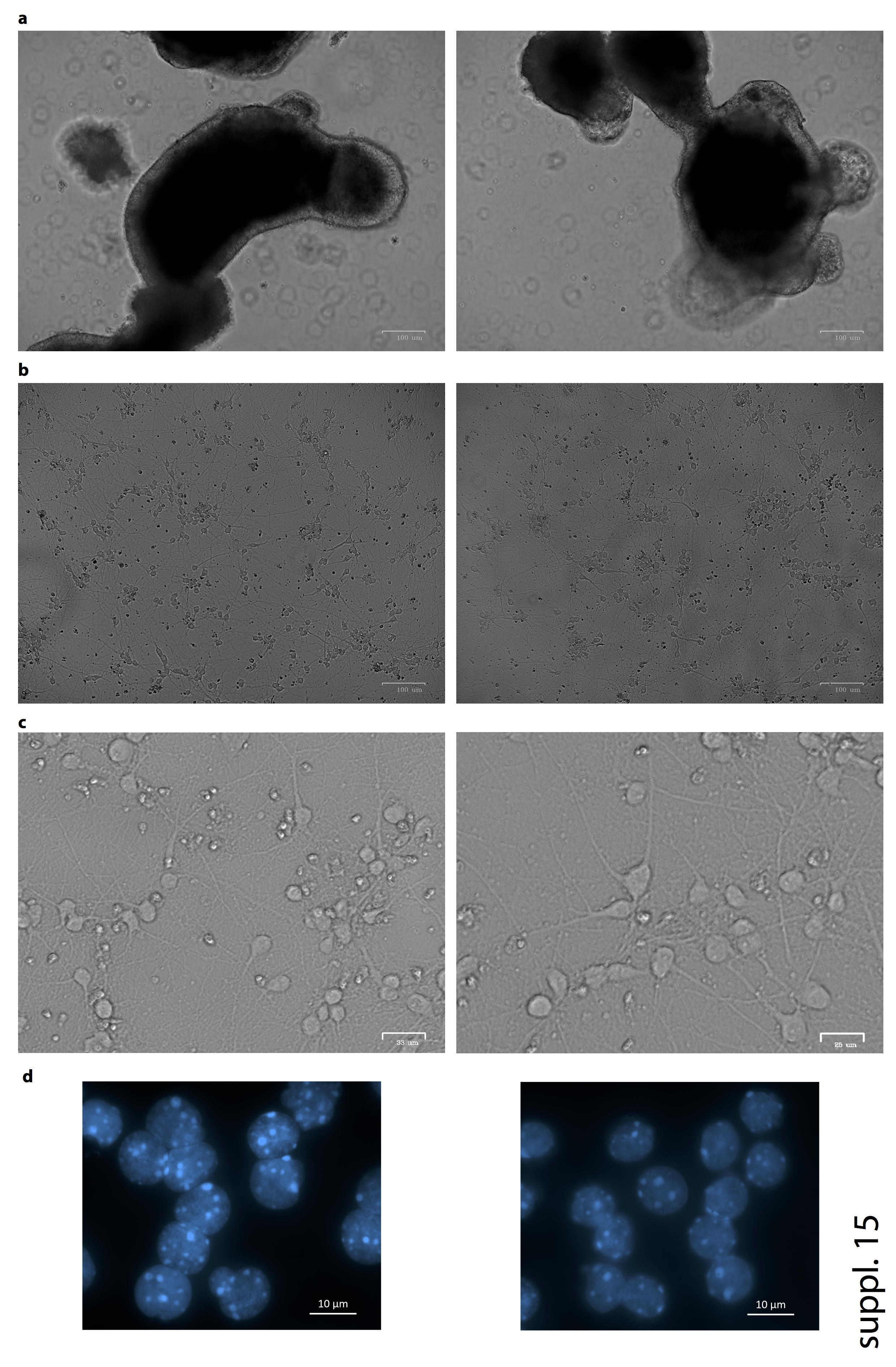

Supplement: S15 Fig — (A) WT (left) and Hmga1 KO (right) embryoid bodies 2 days after retinoic acid addition. No apparent differences can be detected. (B) WT (left) and Hmga1 KO (right) neuronal progenitors at day 6 after plating, which marks the start of synaptic firing [52]. No apparent differences can be detected. (C) WT (left) and Hmga1 KO (right) terminal neurons at day 10 after plating, when pruning has occurred and neurons are mature and stable [52]. No apparent differences can be detected. (D) DAPI staining of nuclei of WT (left) and Hmga1 KO (right) plated neuronal progenitors. No apparent differences can be detected. (TIFF) [file pgen.1007102.s016.tiff]

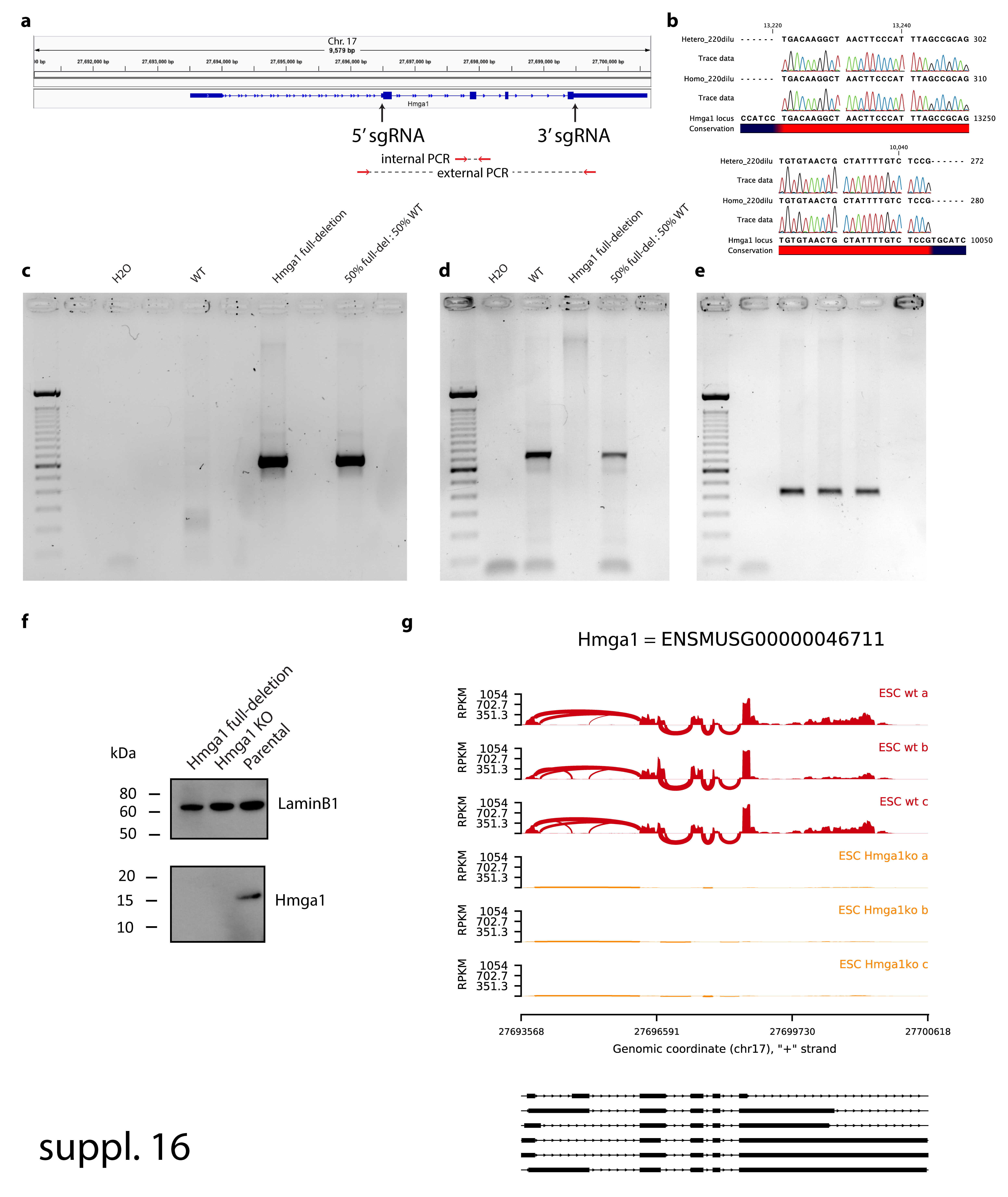

Supplement: S16 Fig — (A) Strategy used for obtaining alleles carrying a “full-deletion” of the coding portion of Hmga1. Indicated are the location of designed sgRNAs and primers used for PCR screening of the single cell clones. Thicker boxes stand for the coding portion of Hmga1 exons. (B) Sequencing results from the PCR product of the “external PCR” (see S16A Fig). Top: 3’ UTR junction. Bottom: 5’ UTR junction. (C-E) PCRs showing presence/absence of respectively, from left to right, the external and internal Hmga1 regions (see S16A Fig) and an unrelated control region (see Materials and methods) for the indicated samples. In the WT background the external PCR (C) is too big to be amplified with the cycling conditions set. Sample loading in (E) is the same as in (D). A 100bp ladder (ThermoFisher, 15628019) was used, with reference bands at 2000, 1500, and 600 bp. (F) WB on the indicated cell lines. Loading control and Hmga1-specific antibody staining highlight absence of Hmga1 in the KO and “full-deletion” clones. (G) Sashimi plot over the Hmga1 locus for WT and Hmga1 “full-deletion” replicates, showing absence of transcription from the deleted locus. (TIF) [file pgen.1007102.s017.tif]

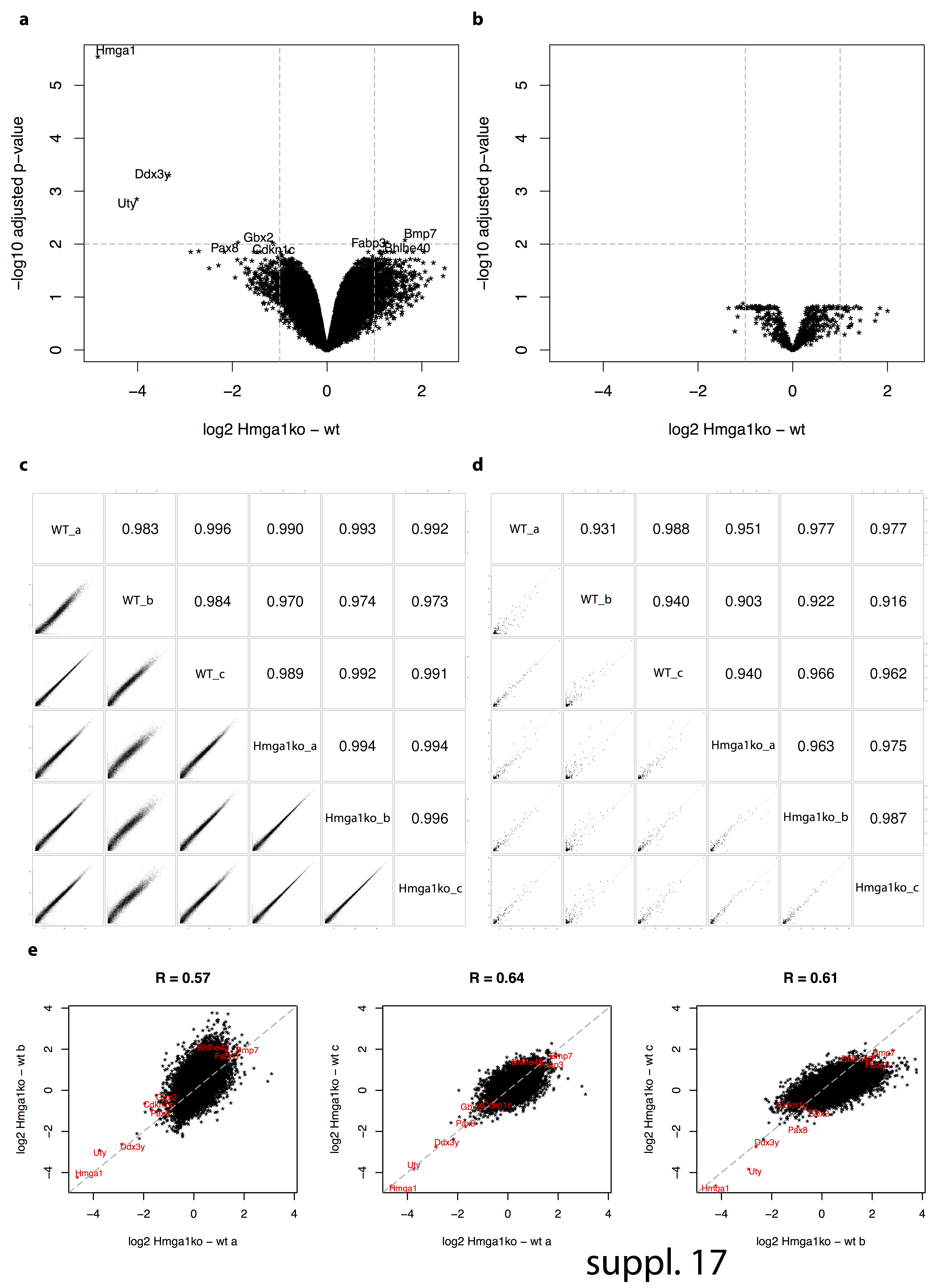

Supplement: S17 Fig — (A) Transcriptomic comparison of Hmga1 full-deletion vs. parental cell line at the gene level. Gene names are indicated if the gene is significantly differentially expressed (adjusted p-value < 0.01 and absolute fold-change of at least 2). (B) Transcriptomic comparison of Hmga1 full-deletion vs. parental cell line at repetitive regions of the genome as defined by RepeatMasker, excluding repeats lying on the same or opposite strand of annotated transcripts (Materials and methods). Repeat elements show no significant changes (adjusted p-value < 0.01 and absolute fold-change of at least 2). Quantification was performed on the level of RepeatMasker repeat “names”. (C)-(D) Scatterplots and Pearson correlations for all RNA-seq samples, illustrating the very high similarity between all samples at genes (C) and the high similarity at repeats (D). (E) Scatterplots showing reproducibility of the transcriptional differences in the pairwise comparisons of WT and KO samples (all sequenced on the same lane). The significantly changing genes are shown in red. (TIFF) [file pgen.1007102.s018.tiff]

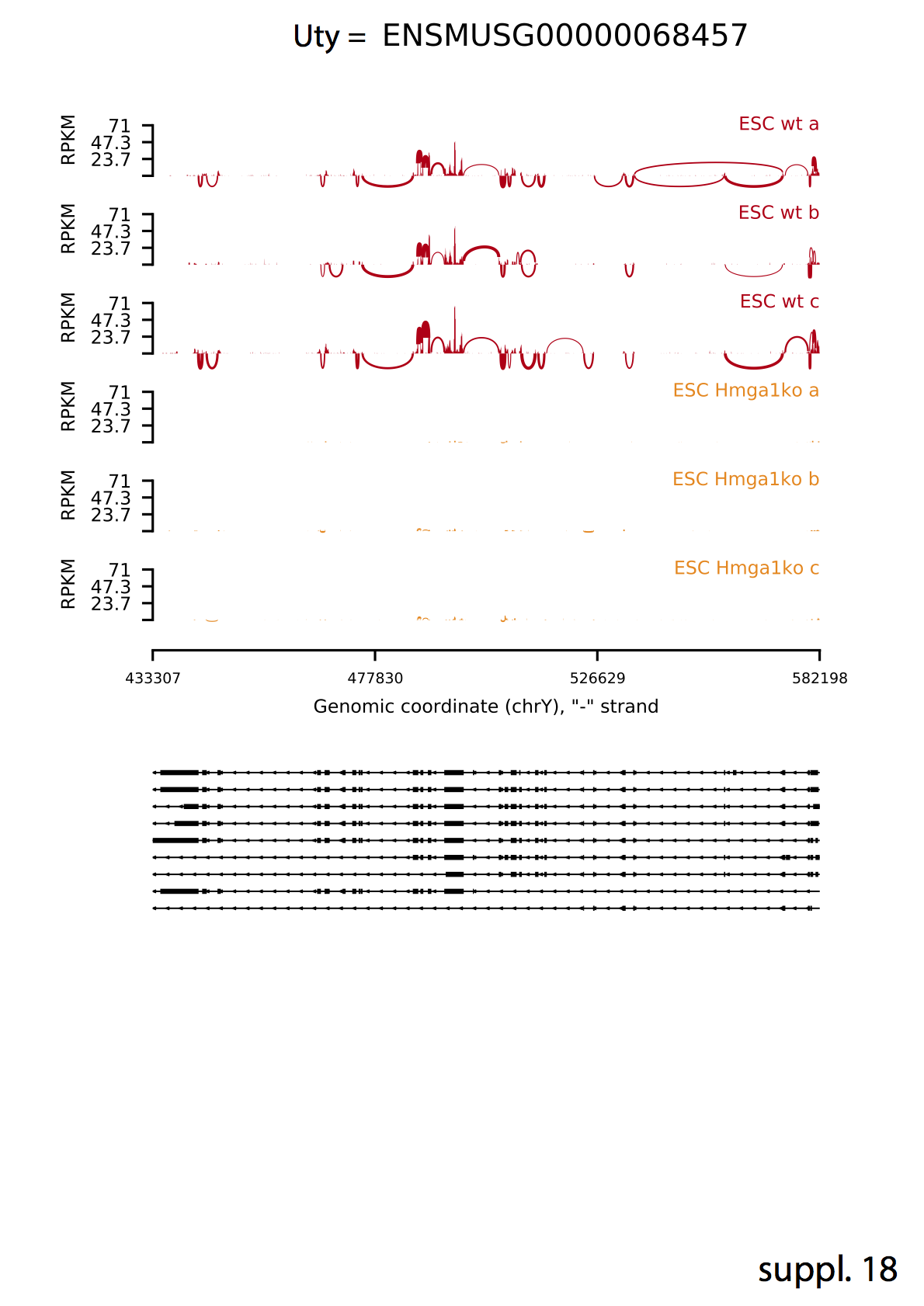

Supplement: S18 Fig — Sashimi plot over the only gene significantly changed in both Hmga1 KO and full-deletion clones. Depicted are reads in the WT and full-deletion condition over the Uty gene, which already in the WT condition is transcribed at very low levels (note the missing exon-exon junctions). For comparison with the expression levels of a transcript that is efficiently translated see S16G. (TIFF) [file pgen.1007102.s019.tiff]
